# Does the Addition of a Collis Gastroplasty to Antireflux Surgery Reduce Hiatal Hernia Recurrence?: A Systematic Review and Meta-Analysis

**DOI:** 10.3390/jcm15103827

**Published:** 2026-05-15

**Authors:** Faith Trinh, Sukhdeep Jatana, Haley Frerichs, Zaharadeen Jimoh, Steffane McLennan, Armin Rouhi, Janice Y. Kung, Vickie Ringuette, Uzair Jogiat, Simon Turner, Daniel Birch, Noah J. Switzer, Shahzeer Karmali

**Affiliations:** 1 Department of Surgery, University of Alberta, Edmonton, AB T6G 2B7, Canadasjatana@ualberta.ca (S.J.); rouhi@ualberta.ca (A.R.);; 2 Faculty of Medicine and Dentistry, University of Alberta, Edmonton, AB T6G 2R7, Canada; 3 Faculty of Science, University of Alberta, Edmonton, AB T6G 2E9, Canada; 4 Geoffrey and Robyn Sperber Health Sciences Library, University of Alberta, Edmonton, AB T6G 1C9, Canada; 5 Centre for Advancement of Surgical Education and Simulation (CASES), Royal Alexandra Hospital, Edmonton, AB T5H 3V9, Canada

**Keywords:** Collis gastroplasty, hiatal hernia, shortened esophagus, gastroesophageal reflux disease, fundoplication

## Abstract

**Introduction:** The role of Collis gastroplasty has traditionally been reserved for patients with a shortened esophagus due to chronic gastroesophageal reflux disease (GERD). However, its necessity has been questioned, leading to a decline in popularity. This systematic review and meta-analysis aimed to evaluate the efficacy of hiatal hernia repair with fundoplication, with versus without Collis gastroplasty. **Methods:** A systematic search of Ovid MEDLINE, Ovid Embase, Scopus, Web of Science Core Collection, and the Cochrane Library (via Wiley) was performed in May 2025. Studies were included if they compared outcomes or the safety profile of Collis gastroplasty versus no Collis gastroplasty during fundoplication for hiatal hernia repair. Meta-analyses were conducted using a random-effects model and restricted maximum likelihood. **Results:** Of 664 unique results, 17 studies comprising 4048 patients were included. There was a female predominance (65.4%), with a weighted mean age of 58.9 ± 14.0 years and follow-up of 43.5 ± 43.1 months. Patients who underwent Collis gastroplasty represented 35.8% of the cohort. Nissen fundoplication was the most common procedure in both the Collis (91.9%) and non-Collis (84.5%) groups. Most studies had selection bias, in which only patients who did not have sufficient intraoperative intra-abdominal esophageal length underwent Collis gastroplasty. Recurrence rates were similar (13.5% vs. 13.2%). Collis gastroplasty was not associated with a reduction in hiatal hernia recurrence (OR 0.53, 95% CI 0.23–1.22). Symptom outcomes, including regurgitation (OR 0.53, 95% CI 0.05–5.39), reflux (OR 0.81, 95% CI 0.03–22.12), dysphagia (OR 1.12, 95% CI 0.62–2.04), and use of antireflux medication on follow-up (OR 1.15, 95% CI 0.62–2.15), were not significantly different. However, Collis gastroplasty was associated with a higher risk of complications, including overall complications (OR 2.63, 95% CI 1.55–4.46), leak (OR 3.35, 95% CI 1.11–10.05), and surgical site infection (OR 8.28, 95% CI 1.16–59.10). There were no significant differences in abscess formation (OR 5.97, 95% CI 0.77–46.49), length of stay (mean difference 0.36 days, 95% CI −0.30 to 1.01), readmission (OR 1.13, 95% CI 0.36–3.60), reoperation (OR 1.24, 95% CI 0.64–2.41), or mortality (OR 1.08, 95% CI 0.45–2.57). **Conclusions:** Collis gastroplasty was not associated with a decreased risk of hiatal hernia recurrence or improvement in other efficacy measures, but this is in the context of a strong component of selection bias. In this context, there may be a role for Collis gastroplasty in difficult cases if the rate of recurrence does not differ from those with sufficient length, but this must be balanced against a significantly increased risk of complications.

## 1. Introduction

The short esophagus is described as one of the anatomic challenges of antireflux surgery (ARS). It is defined as a gastroesophageal junction which cannot be reduced to greater than 2.5 cm below the hiatus without tension [1]. It most often originates from chronic inflammation in the context of gastroesophageal reflux disease (GERD) [1]. The consequent fibrotic scarring can produce a short esophagus. While some apparently short esophagi can be intra-abdominally reduced with surgical mobilization and high mediastinal dissection, some are resistant to this, even with extensive mobilization. In these cases, an esophageal lengthening operation known as Collis is employed.

Collis gastroplasty was first described in 1957 [2] and has since been combined with fundoplication as an antireflux operation for patients with short esophagi [1]. This approach has been associated with impressive patient postoperative satisfaction, achieving as high as a 100% satisfaction rate in a study by Perrone et al. [3]. In patients with giant paraesophageal hernias, Collis gastroplasty with fundoplication was associated with lower rates of postoperative epigastric pain, postprandial bloating, and shortness of breath relative to those who underwent fundoplication alone [4].

Despite its efficacy, there are concerns that Collis gastroplasty poses an increased risk of perioperative leak due to the creation of a new staple line [1]. There are also concerns that the distal esophagus, now composed of acid-producing gastric mucosa, lacks typical motility and is associated with an increased risk of esophagitis [1]. Indeed, in a retrospective review by Lu et al. [5], there was a subset of patients who underwent Collis gastroplasty who were found to have ulcerative lesions in their neoesophagus, requiring postoperative medical antireflux therapy. Furthermore, aggressive laparoscopic mediastinal dissection has been discussed as an effective alternative to Collis gastroplasty in patients with moderately short esophagi [6]. As such, Collis gastroplasty has somewhat fallen out of favor.

The impact of Collis gastroplasty on hernia recurrence remains unclear, with heterogeneous outcomes reported across contemporary series and no consensus on its routine use. The aim of this systematic review and meta-analysis is to compare the recurrence rate of fundoplication for hiatal hernias with and without Collis gastroplasty.

## 2. Methods

### 2.1. Literature Review and Inclusion Criteria

On 20 May 2025, a comprehensive search of Ovid MEDLINE, Ovid Embase, Scopus, Web of Science Core Collection, and Cochrane Library (via Wiley) was performed in collaboration with a medical librarian (Supplementary Materials S1 and S2). Studies were included if they included an adult population (≥18 years old), compared subgroups of fundoplication with Collis gastroplasty vs. fundoplication alone, had outcomes presented as binomial data, and were either a case series, cohort study, case–control study, or randomized controlled trial (RCT). Studies were excluded if they included fewer than 5 patients, studied pediatric patients (<18 years old), or were non-comparative studies. Studies were also excluded that were published before the year 2000 to present a contemporary review. This study was reported in accordance with the PRISMA 2020 checklist for systematic reviews (Supplementary Material S3).

### 2.2. Study Selection

The Covidence deduplication feature was used to initially remove duplicates, and the remaining duplicates were removed manually. Title and abstract screening were performed by two independent authors and discrepancies were resolved by a third author. Full-text articles were then reviewed by two independent authors according to the inclusion and exclusion criteria. Discrepancies were then resolved by a senior author.

### 2.3. Data Collection

Data extraction was performed in duplicate and verified by a senior author. Data collected included the type of fundoplication used, surgical approach (minimally invasive vs. laparotomy vs. thoracotomy), use of mesh, use of a diaphragm-relaxing incision, and follow-up periods. Regarding outcomes, data were collected on the rate of recurrence, overall complications, abscess formation, skin and soft tissue infection (SSI), leak, and mortality. Data on the rate of postoperative dysphagia, GERD, use of medical antireflux therapy, and regurgitation were also extracted. Data on the average lengths of stay and reoperation and readmission rates were collected. For studies with concern for overlapping cohorts, the cohort demographics and procedural details of the most recent paper were used, and outcomes from older papers [7] were only used if not reported in the most recent paper [8].

### 2.4. Risk of Bias Assessment

Risk of bias assessment was completed in duplicate using MINORS criteria for all studies [9]. Studies were considered to be of low quality if they had a MINORS score < 13, moderate quality if they had a MINORS score of 14–19, and high quality if they had a MINORS score of 20–24.

### 2.5. Statistical Analysis

Continuous data were presented using means and standard deviations or interquartile range (IQR) where available. Categorical data were presented using absolute numbers and percentages. A meta-analysis with a random-effects model and restricted maximum likelihood was used. Results are presented as odds ratios for categorical outcomes and as mean differences for continuous outcomes; 95% confidence intervals are provided for both. Results are presented in forest plots. Heterogeneity was measured with the *I*^2^ statistic (<50% low, 50–75% moderate, and >75% high). Funnel plots were used to assess publication bias. Egger’s test was used to assess for small study bias. All statistical analyses were performed with STATA18 [10]. We attempted to perform a post hoc subgroup analysis of studies that minimized selection bias (in which there was no significant difference in intraoperative anatomy between groups undergoing Collis or not), but could not, as only one article included met these criteria. Three additional post hoc analyses were performed, analyzing studies published after 2015, as technique and laparoscopy uptake have likely increased, studies that only reported on minimally invasive techniques (excluding studies that did not describe the approach used and any studies that included any proportion of patients with an open surgery), and studies excluding mesh use (if not mentioned, they were included for analysis). An additional post hoc analysis including only studies that described clinical recurrence was not performed due to insufficient information from manuscripts or an insufficient number focusing on clinical recurrence.

For the analysis, the following assumptions or adjustments were made: for Légner et al., the non-Collis cohort consisted of 87 fundoplication operations and 3 without, but demographic information was only available for the whole cohort [11]; for Lugaresei et al., there was a different number available for overall cohort sizes and for complications for each cohort [8]; and some studies also had different denominators for different complications [12,13].

## 3. Results

### 3.1. Study Selection

**Figure 1:**
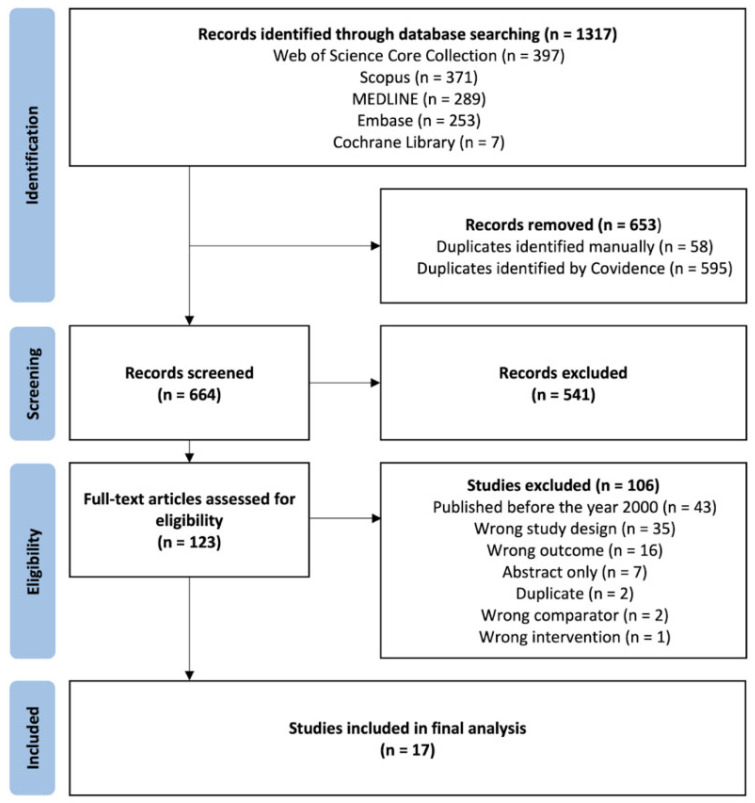
PRISMA flow diagram of study inclusion.

A total of 1317 records were identified, and 653 duplicates were removed. This resulted in a remaining 664 studies, which underwent title and abstract screening. Then, 541 were excluded due to irrelevance to the study objectives. The remaining 123 studies underwent full-text review and 106 were removed as per the exclusion criteria. A total of 17 studies were included in the final analysis [3,4,5,7,8,11,12,13,14,15,16,17,18,19,20,21,22]. These included one prospective cohort study, 15 retrospective cohort studies, and one retrospective case–control study. Please see Figure 1 [23].

### 3.2. Description of Population Cohort and Techniques

A total of 4048 participants were included with a weighted mean age of 58.9 +/− 14.0 years and a mean follow-up of 43.5 +/− 43.1 months. In total, 2649 females were included (65.4%). A total of 1451 participants underwent Collis gastroplasty (35.8%). There was a total of 3561 Nissen fundoplications (88.0%) and 440 partial fundoplications (10.9%), including six Dor fundoplications (0.1%), 288 Toupet fundoplications (7.1%), 22 Belsey Mark IV fundoplications (0.5%), and 16 shortened anterior segment fundoplications (0.4%). Mesh was used in 226 patients (5.6%) and a diaphragm-relaxing incision was used in one case. For 47 participants, the type of fundoplication was not specified. Please see Table 1 and Supplementary Material S4 for details.

### 3.3. Comparison of Efficacy Measures of Collis vs. Non-Collis Cohorts

**Figure 2:**
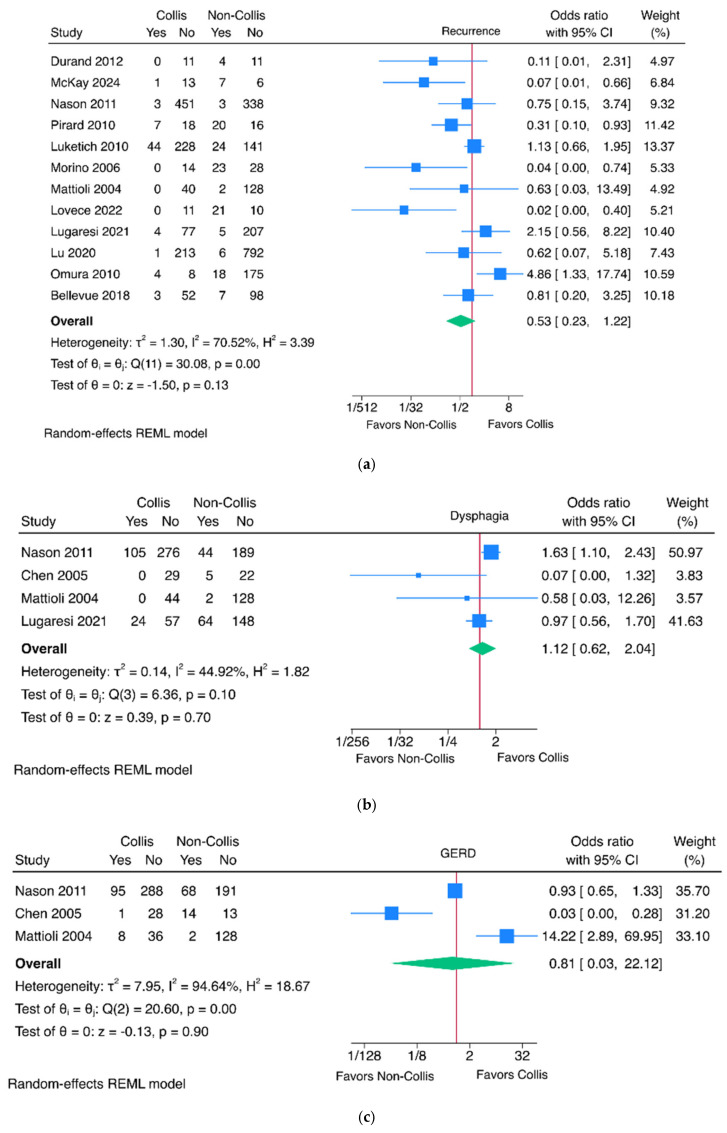

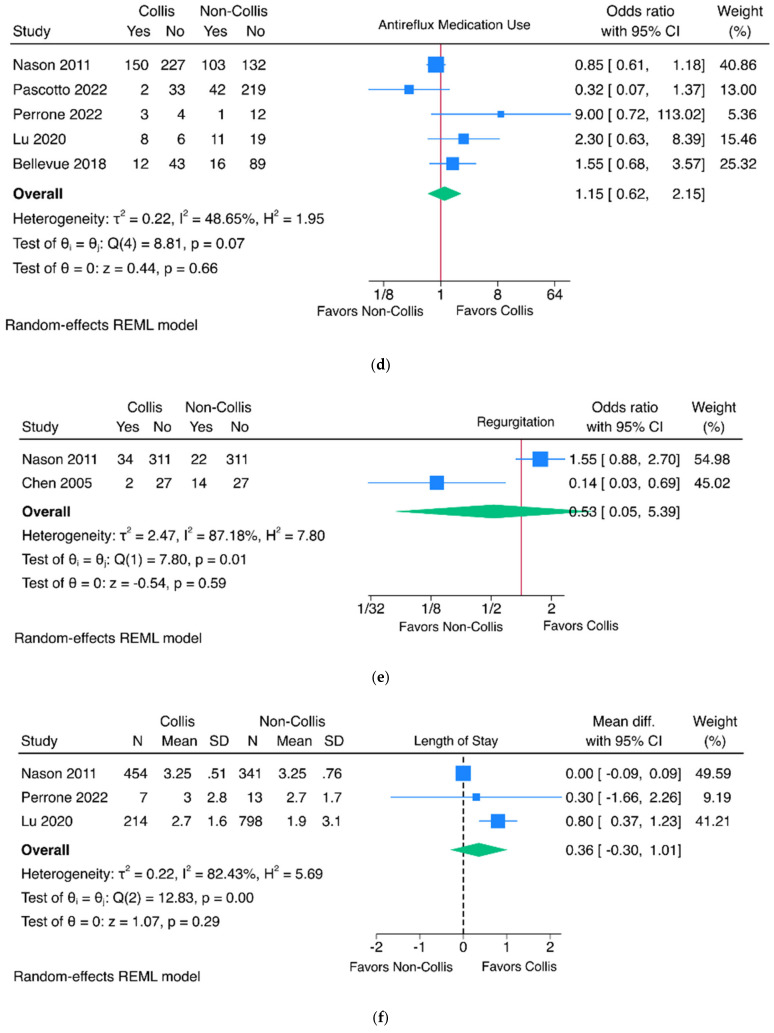
Comparative meta-analysis of efficacy measures of fundoplication with and without Collis gastroplasty. Outcomes assessed include hiatal hernia recurrence [4,5,8,12,13,14,16,17,18,19,20,21] (**a**), postoperative dysphagia [4,8,15,18] (**b**), gastroesophageal reflux disease [4,15,18] (**c**), need for medical antireflux therapy [3,4,5,14,22] (**d**), regurgitation [4,15] (**e**), and length of stay [3,4,5] (**f**).

There was no significant difference in the rate of recurrence between the groups (12 studies, OR 0.53, 95% CI 0.23–1.22, *p* = 0.13) (Figure 2a). Regarding symptom management, there was no significant difference in the rates of postoperative dysphagia (four studies, OR 1.12, 95% CI 0.62–2.04, *p* = 0.70) (Figure 2b), GERD (three studies, OR 0.81, 95% CI 0.03–22.12, *p* = 0.90) (Figure 2c), need for medical antireflux therapy (five studies, OR 1.15, 95% CI 0.62–2.15, *p* = 0.66) (Figure 2d), or regurgitation (two studies, OR 0.53, 95% CI 0.05–5.39, *p* = 0.59) (Figure 2e). Furthermore, there was no statistically significant difference in the mean lengths of stay between the groups (three studies, MD 0.36, 95% CI −0.30–1.01, *p* = 0.29) (Figure 2f). Funnel plots are available in Supplementary Material S5.

### 3.4. Safety Outcomes of Collis vs. Non-Collis Cohorts

**Figure 3:**
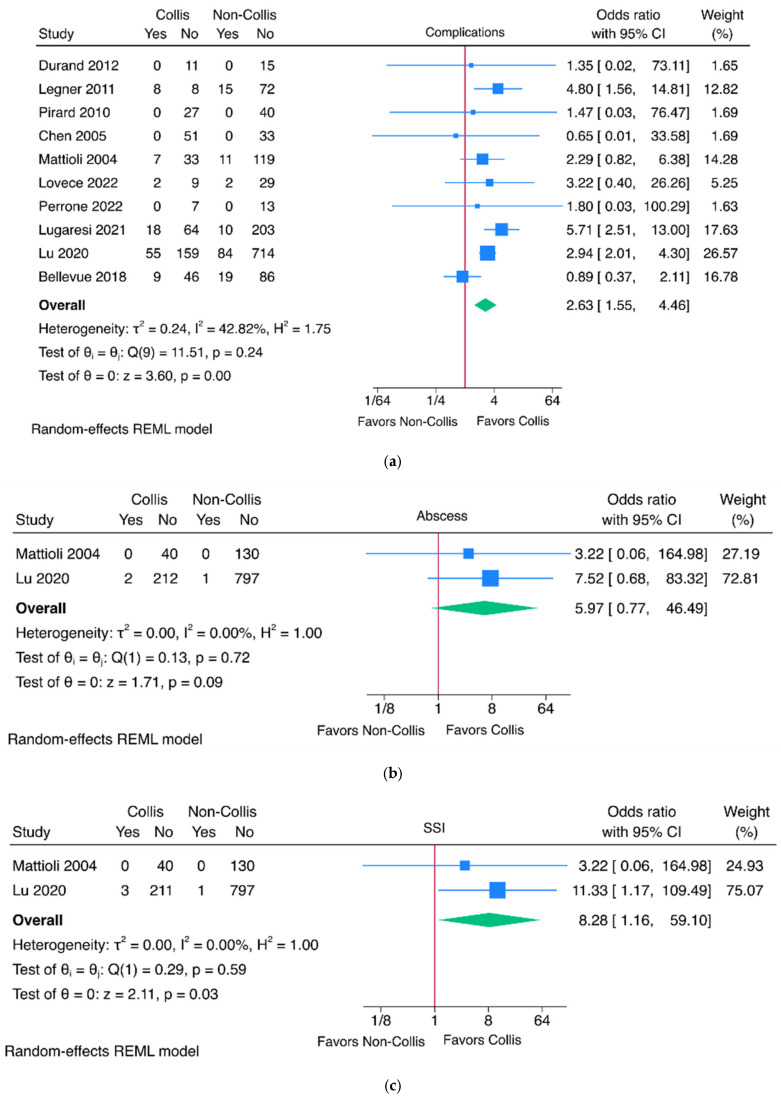

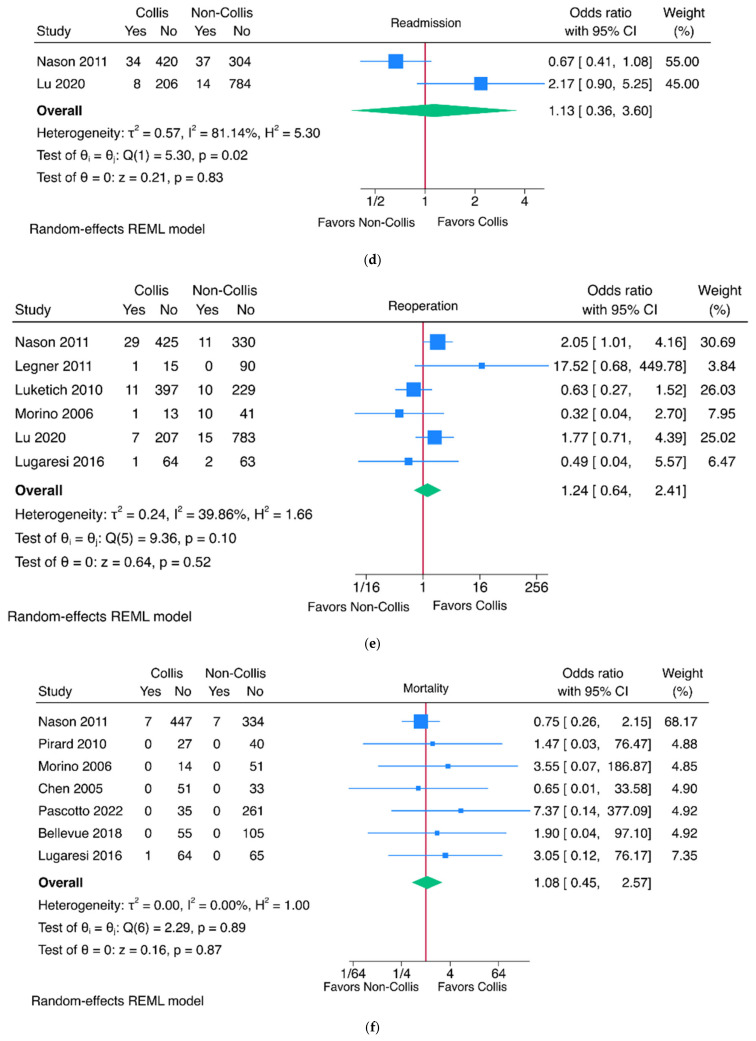

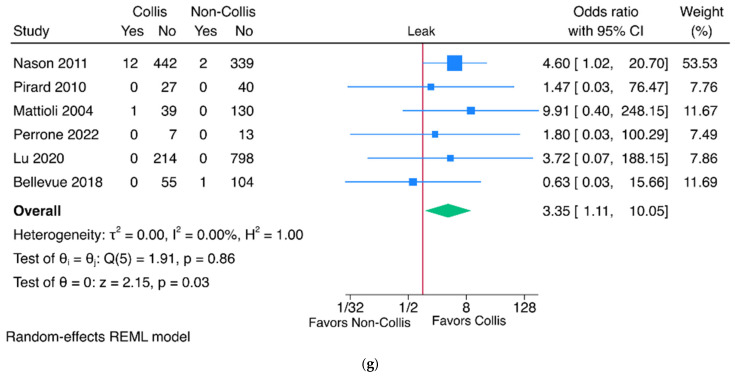
Comparative meta-analysis of safety measures of fundoplication with and without Collis gastroplasty. Outcomes assessed include overall complications [3,5,8,11,13,14,15,16,17,18] (**a**), abscess formation [5,18] (**b**), surgical site infection [5,18] (**c**), readmission [4,18] (**d**), reoperation [1,5,7,11,12,20] (**e**), mortality [4,7,13,14,15,20,22] (**f**), and leak [3,4,5,13,14,18] (**g**). For complications, for Lugaresei 2021 [8], the percentage of complications in each group was used, with 311 total patients, but previous tables show a total of 313 patients and 315 patients; the denominator used for these calculations was 315 patients.

The rate of overall complications was significantly higher in the Collis gastroplasty group (10 studies, OR 2.63, 95% CI 1.55–4.46, *p* < 0.01) (Figure 3a). There was no significant difference in abscess formation (two studies, OR 5.97, 95% CI 0.77–46.49, *p* = 0.09) (Figure 3b). The rate of SSI was significantly higher in the Collis group (two studies, OR 8.28, 95% CI 1.16–59.10, *p* = 0.03) (Figure 3c). There was no significant difference in the rate of readmission (two studies, OR 1.13, 95% CI 0.36–3.60, *p* = 0.83) (Figure 3d), reoperation (six studies, OR 1.24, 95% CI 0.64–2.41, *p* = 0.52) (Figure 3e), or mortality (seven studies, OR 1.08, 95% CI 0.45–2.57, *p* = 0.87) (Figure 3f) between groups. The rate of leaks was significantly greater in the Collis gastroplasty group (six studies, OR 3.35, 95% CI 1.11–10.05, *p* = 0.03) (Figure 3g). Funnel plots are available in Supplementary Material S6.

### 3.5. Studies with Low Selection Bias

In our review, we identified one study with low selection bias. In a retrospective cohort study by Chen et al. [15], participants who underwent antireflux repair for Barrett’s esophagus between January 1976 and January 1999 were included. In total, 33 patients underwent a Nissen fundoplication prior to 1990. After this point, Collis gastroplasty was introduced and the subsequent 51 participants had this procedure. The Nissen and Collis–Nissen groups were comparable in age (48.8 years vs. 53.2 years, *p* = 0.109), sex (78.8% male vs. 80.4% male, *p* = 0.858), symptom duration (10.4 years vs. 10.0 years, *p* = 0.284), weight (73.4 kg vs. 78.3 kg, *p* = 0.404), and height (167.6 cm vs. 172.3 cm, *p* = 0.352). Furthermore, there were no significant differences in the incidences of preoperative symptoms between the Nissen and Collis–Nissen cohorts with respect to heartburn (78.8% vs. 90.2%, *p* = 0.402), regurgitation (90.9% vs. 90.2%, *p* = 0.786), dysphagia (18.2% vs. 29.4%, *p* = 0.277), or odynophagia (21.2% vs. 37.3%, *p* = 0.120).

Participants in the Nissen cohort were found to have higher rates of heartburn (36.7% vs. 0.0%, *p* < 0.001) and regurgitation (30.3% vs. 2.0%, *p* < 0.001) at 5–10 years postoperation relative to those in the Collis–Nissen cohort. These differences persisted at the 5–10-year postoperative follow-up. In addition, participants in the Nissen cohort were found to have higher rates of dysphagia (18.5% vs. 0.0%, *p* = 0.050) and odynophagia (18.5% vs. 0.0%, *p* = 0.050) at the 5–10-year postoperative follow-up compared with the Collis–Nissen cohort. The authors report no postoperative mortality or major esophageal complications; however, they did not report on specific postoperative safety outcomes.

### 3.6. Sensitivity Analyses

Three sensitivity analyses were performed for our primary outcome of recurrence. The first included only studies after 2015. The results of this analysis were the same as the primary analysis, with no difference in recurrence rates (five studies, OR 0.37, 95%CI 0.08–1.73, *p* = 0.21; Supplementary Material S7). A second sensitivity analysis was performed for studies only including MIS cohorts. The result of this analysis was also the same, showing no difference in recurrence for patients undergoing Collis gastroplasty versus the non-Collis cohort (seven studies, OR 0.32, 95%CI 0.08–1.27, *p* = 0.11); Supplementary Material S8). A third sensitivity analysis was performed only for studies that did not mention mesh use, which showed no difference as well (six studies, OR 0.95, 95%CI 0.33–2.74, *p* = 0.92; Supplementary Material S9).

### 3.7. Risk of Bias and Heterogeneity

According to the MINORS tool, there were two low-quality studies, 14 moderate-quality studies, and one high-quality study (Supplementary Material S10).

Heterogeneity, measured by *I^2^*, was low for outcomes such as dysphagia (44.92%) and antireflux medication use (48.65%); moderate for outcomes such as recurrence (70.52%); and high for GERD (94.64%), regurgitation (87.18%), and length of stay (82.43%). For complications, heterogeneity was low for complications (42.82%), abscess (0.00%), SSI (0.00%), reoperation (39.86%), mortality (0.00%) and leak (0.00%), and high for readmission (81.14%). For sensitivity analysis, heterogeneity was moderate for the post-2015 publication date analysis (70.39%), high for the minimally invasive only analysis (76.33%), and moderate for the non-mesh only cohort (61.82%).

Publication bias was measured by funnel plots and Egger’s test (Supplementary Materials S5–S9). For the primary analyses, Egger’s test was significant (*p* < 0.05), suggesting small study bias, for recurrence. It could not be calculated as convergence was not reached for abscess or regurgitation. For sensitivity analyses for recurrence, Egger’s test was significant for the post-2015 publication date analysis and minimally invasive only analysis.

### 3.8. GRADE Statements

1. The addition of Collis gastroplasty to a fundoplication may not be associated with a decreased risk of recurrence of hiatal hernia (low-certainty evidence).
  a. This is secondary to a significant component of selection bias present in studies, along with moderate–high heterogeneity.
2. The addition of Collis gastroplasty to a fundoplication probably increases the risk of all complications (moderate-certainty evidence).
  a. This is secondary to most studies being of an observational nature and the selection bias associated with those who underwent Collis gastroplasty, likely representing higher risk dissections for shorter esophagi.

## 4. Discussion

This systematic review and meta-analysis provide an updated review of the literature on rates of recurrence and postoperative complications in patients who undergo Collis gastroplasty with fundoplication versus those who undergo fundoplication alone. We found no significant difference in the rates of recurrence between the groups in the context of the heavy selection bias against the efficacy of Collis gastroplasty. The Collis gastroplasty group had a significantly higher rate of overall complications, notably leak. With respect to postoperative symptom management, there was no significant difference in the rates of postoperative dysphagia, GERD, or need for medical antireflux therapy. Many outcomes and complications assessed had limited information available, and as such, meta-analyses with fewer than five studies should be considered exploratory, such as those for dysphagia, GERD, regurgitation, length of stay, abscess formation, SSI, and readmission. This suggests that Collis gastroplasty is not associated with reduced recurrence rates or improved postoperative outcomes; however, the results of this review could be interpreted, as in patients with insufficient intra-abdominal esophageal length, Collis provides comparable results to those who undergo fundoplication with sufficient length. Further, Collis is associated with an increased risk of complications in the context of being performed for more difficult dissections and the generation of a neoesophagus.

To our knowledge, this is the first systematic review and meta-analysis comparing rates of recurrence and other postoperative outcomes between patients undergoing Collis gastroplasty with fundoplication versus fundoplication alone. This systematic review and meta-analysis found no significant difference in recurrence rates between the Collis and non-Collis cohorts. This is consistent with the findings of Lu et al. [5], which reported overall low and comparable hiatal hernia recurrence rates in the Collis and non-Collis groups (0.5% vs. 0.8%, *p* = 0.656). Nason et al. [4] also reported similar radiographic hernia recurrence rates in their Collis vs. non-Collis cohorts (16.6% vs. 19.7%, *p* = 0.353). Interestingly, a number of studies did report lower rates of recurrence in the Collis gastroplasty group relative to the non-Collis group. In a recent study by McKay et al. [19], there was a 7% paraesophageal hernia recurrence rate in the Collis cohort compared to a 54% recurrence rate in the non-Collis group (*p* = 0.008) at the 5-year follow-up. As low as a 0% recurrence rate post-Collis gastroplasty has been reported [17,20]. The high standard deviation of follow-up duration in this study can affect recurrence rate detection, as longer follow-up is more likely to detect recurrence.

Regarding safety outcomes, the findings of our study are consistent with a study by Légner et al. [11], which found a significantly higher postoperative complication rate in patients who underwent Collis gastroplasty (50 vs. 17.7%). Similarly, Lugaresi et al. [7] found that Collis gastroplasty was associated with a significantly higher rate of overall mortality and morbidity. A subgroup analysis revealed that there was a significantly higher rate of morbidity and mortality in the first 32 cases of Collis–Nissen compared to the first 32 cases of standard Nissen fundoplication. Interestingly, this difference was not seen when the morbidity and mortality rates were compared between the last 33 cases of Collis–Nissen and standard Nissen fundoplication. Furthermore, a retrospective study by Zehetner et al. [24] reported no leak or abscess or fistula formation in patients who underwent Collis gastroplasty. The authors proposed that their laparoscopic approach allowed for clearer visualization of the lesser curvature vasculature, therefore allowing them to avoid Collis gastroplasty in patients at high risk of ischemia [25]. Overall, this may suggest that greater experience with Collis gastroplasty in conjunction with predominantly laparoscopic approaches may decrease the complication rate of Collis gastroplasty. Further, consideration of intraoperative leak testing with endoscopy, methylene blue instillation or air leak testing may be of use in difficult dissections. The results of this review also showed wide confidence intervals in certain complications, such as SSI, which may be influenced by a few events and should be interpreted with caution.

A notable consideration in these data is that most of the studies had a significant component of selection bias, where Collis gastroplasty was only performed when there was insufficient intra-abdominal esophageal length. Collis gastroplasty is often used as a last resort operation for the most challenging cases, particularly those involving shortened esophagi, larger hernias, recurrent hernias, and more mediastinal fibrosis. Because Collis gastroplasty is typically reserved for these anatomically more complex cases, the comparable recurrence rates observed in this analysis may suggest that Collis gastroplasty mitigates the increased recurrence risk associated with shortened esophagus rather than indicating equivalent efficacy. Only one study seemed to minimize selection bias, whose result showed improved symptom outcomes in the Collis–Nissen cohort relative to the Nissen cohort [15]. However, we note that the authors did not report on recurrence rates or specific safety outcomes. There was also significant heterogeneity in the definition of recurrence and patient groups; this is noted by the higher heterogeneity scores for the primary and sensitivity analyses for recurrence, as well as the statistically significant Egger’s tests. This weakens the strength of this finding, and future prospective studies should control for technique and patient factors. Nonetheless, Collis may remain a tool in the arsenal of a thoracic or upper gastrointestinal surgeon for difficult procedures. The frequency of Collis is decreasing, which is likely due to better laparoscopic experience and dissection techniques, which further decreases surgeon volume and comfort for this technique. Furthermore, it has a known worse safety profile shown in this review, and thus, Collis gastroplasties will likely be reserved for select cases.

Given its rarity, additional skills training for this procedure may be provided to ensure surgeons have sufficient exposure to employ the Collis gastroplasty when clinically indicated. Lugaresi et al. explicitly discuss the learning curve in their study between the first and second halves of their cohort [7]. While credentialing is not routinely enforced [24], surgeons who would like to have the Collis gastroplasty in their arsenal would likely need a minimally invasive or foregut fellowship; additional exposure through courses organized by national or international minimally invasive surgery organizations can supplement low volumes seen during residency or fellowship. Another field of interest is robotic hiatal hernia repair, with current data suggesting equivalency in outcomes but with the known benefit of enhanced visualization. Only one study has reported on this in the current cohort [3]. While it has been described in the literature, there has been limited data published on robotic Collis procedures, which would be of interest with the growing availability and training of the robot [26,27].

We also recognize some of the limitations and potential sources of bias in this study. One of the weaknesses is in the inclusion of complications from both open and laparoscopic approaches to fundoplication and Collis gastroplasty. For this, we performed a sensitivity analysis excluding open procedures. A sensitivity analysis could not be performed for patients with only clinical recurrence, which may hold more clinical significance than radiologic recurrence alone; the definition of recurrence also varied across the studies. There is also a lack of consistent reporting on patient demographics and medical comorbidities as well as inconsistent selection criteria between studies. As such, it can be challenging to determine whether outcomes are associated with Collis gastroplasty or are derived from baseline differences in the severity of disease between the groups. This also limited any incorporation of patient baseline comorbidities or disease state into the outcome analysis. Further, the lack of reporting of some studies on types of fundoplication and the percentage of recurrent hernias is significant, as it can significantly impact some of the outcomes presented. Another limitation is that this study did not collect information on the development of ulcers in the neoesophagus from retained parietal cells, which may lead to clinically significant outcomes. Postoperative testing of the neoesophagus shows gastric oxyntic mucosa in all patients and acid secretion in patients with abnormal DeMeester scores [28]. This suggests a role of postoperative surveillance in these patients but is beyond the scope of the manuscript, especially of clinically significant outcomes such as esophagitis, ulceration and Barrett’s metaplasia. Furthermore, there were no RCTs comparing Collis gastroplasty with fundoplication with fundoplication alone. The majority of the studies included were retrospective cohort studies, which pose a greater risk of bias. Indeed, in a retrospective cohort study by Lu et al. [5], the Collis gastroplasty group was significantly older and more comorbid than their non-Collis gastroplasty counterparts. Furthermore, they were more likely to have undergone previous revision antireflux surgeries. Future study trials should consider large, prospective, multi-center registries with standardized definitions for radiographic and clinical recurrence.

As the first systematic review and meta-analysis comparing Collis versus non-Collis outcomes, this systematic review can give insights into techniques, efficacy and safety outcomes associated with the Collis gastroplasty. While it will likely remain a niche technique, the outcomes of this review will have to be interpreted in the context of the inherent selection bias of many of the included retrospective studies.

## 5. Conclusions

Collis gastroplasty was not associated with a reduction in recurrence rates or improved postoperative symptom outcomes. It was, however, associated with higher rates of complications, specifically skin and soft tissue infections and postoperative leaks. These findings should be interpreted in the context of the selection bias present in many included studies and comparable rates between the higher-risk Collis cohort and the fundoplication-only cohort. The Collis gastroplasty will have a niche role and its use must be balanced against its risk profile.

## Figures and Tables

**Table 1:** Studies included in the final analysis. The number of participants who underwent each of the following types of fundoplication are indicated when reported.

Study	Single vs. Multicenter (Country)	Design	Participants (N)	Female (%)	Collis (N)				Non-Collis (N)				Primary (%)	Recurrent (%)
					Nissen (N)	Toupet (N)	Belsey Mark IV (N)	Dor (N)	Nissen (N)	Toupet (N)	Belsey Mark IV (N)	Dor (N)		
McKay et al., 2024 [19]	Single (USA)	Prospective cohort	27	74.07%	14				13				100%	0%
					3	11	0	0	4	9	0	0		
Pascotto et al., 2022 [22]	Single (Belgium)	Retrospective cohort	296	57.09%	35				261				78.7%	21.3%
									261	0	0	0		
Lovece et al., 2022 [17]	Single (Italy)	Retrospective cohort	42	54.76%	11				31				0%	100%
					5	6	0	0	12	19	0	0		
Perrone et al., 2022 [3]	Single (USA)	Retrospective cohort	20	45.00%	7				13				Not specified	
								
Lugaresi et al., 2021 [8]	Single (Italy)	Retrospective cohort	315	62.06%	82				213				100%	0%
					82	0	0	0	213	0	0	0		
Lu et al., 2020 [5]	Multicenter (USA)	Retrospective cohort	1012	71.25%	214				798				79.9%	20.1%
					210	4	0	0	783	15	0	0		
Bellevue et al., 2018 [14]	Single (USA)	Retrospective case–control	160	54.38%	55				105				100%	0%
					55	0	0	0	105	0	0	0		
Lugaresi et al., 2016 [7]	Single (Italy)	Retrospective cohort	130	41.54%	65				65				Not specified	
					65	0	0	0	65	0	0	0		
Durand et al., 2012 [16]	Single (Argentina)	Retrospective cohort	26	53.85%	11				15				Not specified	
					11	0	0	0	15	0	0	0		
Nason et al., 2011 [4]	Single (USA)	Retrospective cohort	795	75.22%	454				341				Not specified	
								
Légner et al., 2011 [11]	Single (USA)	Retrospective cohort	106	68.87%	16				87				0%	100%
					13	2	1	0	44	34	3	6		
Omura et al., 2010 [21]	Single (Japan)	Retrospective cohort	205	41.95%	12				193				Not specified	
					12	0	0	0	80	113	0	0		
Luketich et al., 2010 [12]	Single (USA)	Retrospective cohort	662	74.77%	408				239				100%	0%
									
Pirard et al., 2010 [13]	Single (Belgium)	Retrospective cohort	67	47.76%	27				40				70.1%	29.9%
					27	0	0	0	40	0	0	0		
Morino et al., 2006 [20]	Single (Italy)	Retrospective cohort	65	55.38%	14				51				Not specified	
					14	0	0	0	51	0	0	0		
Chen et al., 2005 [15]	Single (Canada)	Retrospective cohort	84	20.24%	51				33				91.7%	8.3%
					51	0	0	0	33	0	0	0		
Mattioli et al., 2004 [18]	Single (Italy)	Retrospective cohort	170	44.71%	40				130				100%	0%
					29	0	11	0	123	0	7	0		

## Data Availability

Data was generated through the collection of the published literature. Data is available upon reasonable request to the Corresponding Author.

## Supplementary Materials

The following supporting information can be downloaded at: https://www.mdpi.com/article/10.3390/jcm15103827/s1, Supplementary Material S1: Search protocol; Supplementary Material S2: Literature search; Supplementary Material S3: PRISMA 2020 checklist; Supplementary Material S4: Characteristics of included studies and operative techniques; Supplementary Material S5: Funnel plots for efficacy measures such as recurrence (a), dysphagia (b), GERD (c), use of post-operation anti-reflux medications (d), regurgitation (e), and length of stay (f); Supplementary Material S6: Funnel plots for safety measures such as overall complications (a), abscess formation (b) SSI (c), readmission (d), reoperation (e), mortality (f), and leak (g); Supplementary Material S7: Comparative meta-analysis of recurrence with fundoplication with and without Collis gastroplasty using studies published after 2015 (a); Funnel plot for recurrence using studies published after 2015 (b); Supplementary Material S8: Comparative meta-analysis of recurrence with fundoplication with and without Collis gastroplasty using studies with minimally invasive techniques (a); Funnel plot for recurrence using studies with minimally invasive techniques (b); Supplementary Material S9: Comparative meta-analysis of recurrence with fundoplication with and with Collis gastroplasty using studies that did not use mesh (a). Funnel plot for recurrence using studies that did not use mesh (b); Supplementary Material S10: Risk of bias assessment.
