# Supplementary material for: Does the Addition of a Collis Gastroplasty to Antireflux Surgery Reduce Hiatal Hernia Recurrence?: A Systematic Review and Meta-Analysis
Source: J Clin Med. 2026 May 15;15(10):3827. doi: 10.3390/jcm15103827 (PMC13208049; doi:10.3390/jcm15103827)
Supplement: Supplementary file 1 [file jcm-15-03827-s001.zip › jcm-4231273-supplementary/Supplementary Material 1 - Search Protocol.pdf]

Supplementary Material 1. Search protocol.

Project Title: Evaluation of Collis Gastroplasty on GERD: A Systematic Review

What role would you like the librarian to have in this project?
___ **Instructional** (Librarian will instruct in how to search databases and export results)

_X_ **Co-authorship** (Librarian will work with the team to develop the search strategy, search databases, export results, write search part of the methods section, document search appendix for article, and review the final draft of the article) – see last page for details of librarian co-author responsibilities

PUBLICATION

Are you planning to register your study protocol? If Yes, Name of registry:

No

Are you planning to publish your protocol?

Yes, as a supplementary material alongside the final publication

Where are you planning to publish the final manuscript?

The targeted journal for this publication would be a high level surgical journal aimed at the most up-to-date information on anti-reflux surgery.

PROJECT TIMELINES

1. Meet with Librarian April or May 2025

2. Abstract/Title Review and then Full text review by end of May 2025

3. Data extraction June 2025

4. Data analysis July-August 2025

5. First draft August 2025

6. Completed Manuscript August-September 2025

INTRODUCTION
Describe the Background to this project

Esophageal shortening is a complication of chronic gastroesophageal reflux disease (GERD) which can make esophageal mobilization during paraesophageal hernia repair more challenging.[1] Longitudinal esophageal lengthening procedures including Collis gastroplasty are reported to be safe and effective adjuncts to fundoplication to improve GERD symptoms for patients with a shortened esophagus following complete mediastinal dissection.[1–6] Despite increasing evidence to support the use of Collis gastroplasty, there is concern for persistent acid production in retained parietal cells and associated dysphagia in the amotile neoesphagus and some surgeons recommend aggressive mediastinal dissection over esophageal lengthening.[7]

Objective: This study hopes to provide an up to date evaluation of gastroesophageal reflux disease (GERD) symptoms following anti-reflux surgery with Collis gastroplasty

PICO:

The population of interest will be adult patients with Type I-III hiatal hernias and GERD undergoing anti-reflux surgery with a Collis gastroplasty (CG). The intervention will be CG. Outcomes will include changes to GERD symptoms (as measured with validated questionnaires) at 1+ years following anti-reflux surgery with Collis gastroplasty compared to before surgical intervention and compared to anti-reflux surgery without Collis gastroplasty (NC).

Primary outcome will be GERD scoring prior to CG compared to at 1 year follow up. Secondary outcomes will include comparison of GERD symptoms for CG versus NC, follow up greater than 1 year, and perioperative complications.

Notably, subgroup analysis of randomized controlled trials will be planned with reporting of all primary outcomes in the included comparative studies (i.e. CG versus NC).

Types of studies: We will include all studies evaluating adult (>=18 years old) patients undergoing CG who report GERD quality of life data using validated questionnaires. Only studies reporting the primary outcome will be included in analysis.

Please list any specific inclusion/exclusion criteria
Exclude:

- n < 5
- studied including patients aged 17 and younger (pediatric patients)
- animal studies
- non-comparative studies (i.e. studies not reporting pre-operative scores)
- abstracts
- non-published studies
- non-english studies
- studies published prior to 1990

Key References (List relevant papers that you have already found)

1. Lu R, Addo A, Broda A, et al. Update on the Durability and Performance of Collis Gastroplasty For Chronic GERD and Hiatal Hernia Repair At 4-Year Post-Intervention. *J Gastrointest Surg Off J Soc Surg Aliment Tract*. 2020;24(2):253-261. doi:10.1007/s11605-019-04438-z

2. Mor A, Lutfi R, Torquati A. Esophageal acid-clearance physiology is altered after Nissen-Collis gastroplasty. *Surg Endosc*. 2013;27(4):1334-1338. doi:10.1007/s00464-012-2609-5

3. Garg N, Yano F, Filipi CJ, Mittal SK. Long-term symptomatic outcomes after Collis gastroplasty with fundoplication. *Dis Esophagus Off J Int Soc Dis Esophagus*. 2009;22(6):532-538. doi:10.1111/j.1442-2050.2009.00943.x

4. Nason KS, Luketich JD, Awais O, et al. Quality of life after collis gastroplasty for short esophagus in patients with paraesophageal hernia. *Ann Thorac Surg*. 2011;92(5):1854-1860; discussion 1860-1861. doi:10.1016/j.athoracsur.2011.06.030

5. Durand L, De Antón R, Caracoche M, et al. Short esophagus: selection of patients for surgery and long-term results. *Surg Endosc*. 2012;26(3):704-713. doi:10.1007/s00464-011-1940-6

6. Montcusí B, Jaume-Bottcher S, Álvarez I, et al. 5-Year Collis-Nissen Gastroplasty Outcomes for Type III-IV Hiatal Hernia with Short Esophagus: A Prospective Observational Study. *J Am Coll Surg*. 2023;237(4):596-604. doi:10.1097/XCS.0000000000000785

7. O'Rourke RW, Khajanchee YS, Urbach DR, et al. Extended transmediastinal dissection: an alternative to gastroplasty for short esophagus. *Arch Surg Chic Ill 1960*. 2003;138(7):735-740. doi:10.1001/archsurg.138.7.735
