# Supplementary material for: Does the Addition of a Collis Gastroplasty to Antireflux Surgery Reduce Hiatal Hernia Recurrence?: A Systematic Review and Meta-Analysis
Source: J Clin Med. 2026 May 15;15(10):3827. doi: 10.3390/jcm15103827 (PMC13208049; doi:10.3390/jcm15103827)
Supplement: Supplementary file 1 [file jcm-15-03827-s001.zip › jcm-4231273-supplementary/Supplementary Material 10 - Risk of Bias.docx]

#### **Supplementary Material 4.** Risk of bias assessment using the Methodological Index for Non-Randomized Studies (MINORS) criteria.

| **Study** | **A Clearly Stated Aim** | **Inclusion of Consecutive Patients** | **Prospective Collection of Data** | **Endpoints Appropriate to the Aim of the Study** | **Unbiased Assessment of the Study Endpoint** | **Follow-up Period Appropriate to the Aim of the Study** | **Loss to Follow-up <5%** | **Prospective Calculation of the Study Size** | **An Adequate Control Group** | **Contemporary Groups** | **Baseline Equivalence of groups** | **Adequate Statistical Analysis** | **Total (Quality)** |
| --- | --- | --- | --- | --- | --- | --- | --- | --- | --- | --- | --- | --- | --- |
| Durand et al. 2012 | 1 | 2 | 0 | 2 | 1 | 2 | 2 | 0 | 2 | 2 | 0 | 2 | 16 (M) |
| Nason et al. 2011 | 2 | 2 | 0 | 2 | 1 | 2 | 1 | 0 | 2 | 2 | 0 | 2 | 16 (M) |
| Legner et al. 2011 | 2 | 2 | 2 | 2 | 1 | 2 | 1 | 0 | 0 | 0 | 0 | 2 | 14 (M) |
| Pirard et al. 2010 | 2 | 2 | 0 | 2 | 1 | 2 | 1 | 0 | 2 | 2 | 0 | 2 | 16 (M) |
| Luketich et al. 2010 | 2 | 2 | 0 | 2 | 1 | 2 | 1 | 0 | 1 | 0 | 0 | 2 | 13 (M) |
| Morino et al. 2006 | 2 | 2 | 1 | 2 | 1 | 2 | 0 | 0 | 0 | 0 | 0 | 2 | 12 (L) |
| Chen et al. 2005 | 2 | 2 | 0 | 2 | 1 | 2 | 2 | 0 | 2 | 2 | 2 | 2 | 19 (H) |
| Mattioli et al. 2004 | 2 | 1 | 1 | 2 | 1 | 2 | 1 | 0 | 2 | 0 | 0 | 2 | 14 (M) |
| Lovece et al. 2022 | 2 | 1 | 2 | 2 | 1 | 2 | 2 | 0 | 2 | 2 | 0 | 2 | 18 (M) |
| Pascotto et al. 2022 | 2 | 2 | 0 | 2 | 1 | 2 | 2 | 0 | 0 | 2 | 0 | 2 | 15 (M) |
| Perrone et al. 2022 | 2 | 0 | 0 | 2 | 1 | 0 | 1 | 0 | 2 | 1 | 0 | 2 | 11 (L) |
| Lugaresi et al. 2021 | 2 | 2 | 1 | 2 | 1 | 2 | 2 | 0 | 2 | 2 | 0 | 2 | 18 (M) |
| Lu et al. 2020 | 2 | 1 | 1 | 2 | 1 | 2 | 1 | 0 | 2 | 2 | 0 | 2 | 16 (M) |
| Omura et al. 2010 | 2 | 1 | 0 | 2 | 1 | 1 | 2 | 0 | 1 | 2 | 0 | 2 | 14 (M) |
| Bellevue et al. 2018 | 2 | 2 | 1 | 2 | 1 | 2 | 0 | 0 | 2 | 2 | 0 | 2 | 16 (M) |
| McKay et al. 2024 | 2 | 2 | 2 | 2 | 1 | 2 | 1 | 0 | 1 | 2 | 0 | 2 | 17 (M) |
| Lugaresi et al. 2016 | 2 | 1 | 0 | 2 | 1 | 2 | 1 | 0 | 2 | 2 | 0 | 2 | 15 (M) |

Abbreviations: H, high quality; L, low quality; M, moderate quality; MINORS, methodical index for non-randomized studies; ROB, risk of bias.
