# Supplementary material for: Does the Addition of a Collis Gastroplasty to Antireflux Surgery Reduce Hiatal Hernia Recurrence?: A Systematic Review and Meta-Analysis
Source: J Clin Med. 2026 May 15;15(10):3827. doi: 10.3390/jcm15103827 (PMC13208049; doi:10.3390/jcm15103827)
Supplement: Supplementary file 1 [file jcm-15-03827-s001.zip › jcm-4231273-supplementary/Supplementary Material 2 - Literature Search.pdf]

Supplementary Material 2. Search Strategies

Database	Search Strategy
**MEDLINE**<br><br>Ovid MEDLINE(R) ALL 1946 to May 19, 2025	1. (collis adj2 gastroplasty).mp.<br>2. (Collis adj2 Nissen).mp.<br>3. ((gastroplasty or fundoplication*) and ("short* esophagus" or brachy?esophagus or brachy-?esophagus)).mp.<br>4. ((antireflux surg* or anti-reflux surg* or antireflux procedur* or anti-reflux procedur*) and ("short* esophagus" or brachy?esophagus or brachy-?esophagus)).mp.<br>5. or/1-4<br>6. animals/ not (animals/ and humans/)<br>7. (veterinary or rabbit or rabbits or animal or animals or mouse or mice or rodent or rodents or rat or rats or murine or hamster* or pig or pigs or piglets or swine or porcine or horse* or equine or cow or cows or cattle or bovine or goat or goats or sheep or lambs or ovine or monkey or monkeys or trout or marmoset$1 or canine or dog or dogs or feline or cat or cats or zebrafish).ti.<br>8. 6 or 7<br>9. 5 not 8<br>10. limit 9 to english language
**Embase**<br><br>Ovid Embase 1974 to 2025 May 19	1. (collis adj2 gastroplasty).mp.<br>2. (Collis adj2 Nissen).mp.<br>3. ((gastroplasty or fundoplication*) and ("short* esophagus" or brachy?esophagus or brachy-?esophagus)).mp.<br>4. ((antireflux surg* or anti-reflux surg* or antireflux procedur* or anti-reflux procedur*) and ("short* esophagus" or brachy?esophagus or brachy-?esophagus)).mp.<br>5. or/1-4<br>6. animal/ not (animal/ and human/)<br>7. (veterinary or rabbit or rabbits or animal or animals or mouse or mice or rodent or rodents or rat or rats or murine or hamster* or pig or pigs or piglets or swine or porcine or horse* or equine or cow or cows or cattle or bovine or goat or goats or sheep or lambs or ovine or monkey or monkeys or trout or marmoset$1 or canine or dog or dogs or feline or cat or cats or zebrafish).ti.<br>8. 6 or 7<br>9. 5 not 8<br>10. limit 9 to english language
**Cochrane Library**<br>via Wiley	#1 collis NEAR/2 gastroplasty<br>#2 Collis NEAR/2 Nissen<br>#3 ((gastroplasty or fundoplication*) and ((short* NEXT esophagus) or brachy?esophagus or brachy-?esophagus))<br>#4 (((antireflux NEXT surg*) or (anti-reflux NEXT surg*) or (antireflux NEXT procedur*) or (anti-reflux NEXT procedur*)) and ((short* NEXT esophagus) or (brachy? NEXT esophagus) or brachy-?esophagus))

	#5{OR #1-#4}<br><br>Limit: English language
**Scopus**	(TITLE-ABS-KEY (collis W/2 gastroplasty) OR TITLE-ABS-KEY (collis W/2 nissen) OR TITLE-ABS-KEY ((gastroplasty OR fundoplication*) AND ("short* esophagus" OR brachyesophagus OR brachyoesophagus OR brachy-esophagus OR brachy-oesophagus)) OR TITLE-ABS-KEY (("antireflux surg*" OR "anti-reflux surg*" OR "antireflux procedur*" OR "anti-reflux procedur*") AND ("short* esophagus" OR brachyesophagus OR brachyoesophagus OR brachy-esophagus OR brachy-oesophagus))) AND NOT TITLE (veterinary OR rabbit OR rabbits OR animal OR animals OR mouse OR mice OR rodent OR rodents OR rat OR rats OR murine OR hamster* OR pig OR pigs OR piglets OR swine OR porcine OR horse* OR equine OR cow OR cows OR cattle OR bovine OR goat OR goats OR sheep OR lambs OR ovine OR monkey OR monkeys OR trout OR marmoset* OR canine OR dog OR dogs OR feline OR cat OR cats OR zebrafish) AND (LIMIT-TO (LANGUAGE , "English"))
**Web of Science Core Collection**	(TS= (collis NEAR/2 gastroplasty) OR TS= (collis NEAR/2 nissen) OR TS= ((gastroplasty OR fundoplication*) AND ("short* esophagus" OR brachyesophagus OR brachyoesophagus OR brachy-esophagus OR brachy-oesophagus)) OR TS= (("antireflux surg*" OR "anti-reflux surg*" OR "antireflux procedur*" OR "anti-reflux procedur*") AND ("short* esophagus" OR brachyesophagus OR brachyoesophagus OR brachy-esophagus OR brachy-oesophagus))) NOT TI= (veterinary OR rabbit OR rabbits OR animal OR animals OR mouse OR mice OR rodent OR rodents OR rat OR rats OR murine OR hamster* OR pig OR pigs OR piglets OR swine OR porcine OR horse* OR equine OR cow OR cows OR cattle OR bovine OR goat OR goats OR sheep OR lambs OR ovine OR monkey OR monkeys OR trout OR marmoset* OR canine OR dog OR dogs OR feline OR cat OR cats OR zebrafish)<br><br>Languages: English
**Google Scholar**	(collis gastroplasty OR collis nissen) -animal -rat -rats -mice -mouse
