# Supplementary material for: Does the Addition of a Collis Gastroplasty to Antireflux Surgery Reduce Hiatal Hernia Recurrence?: A Systematic Review and Meta-Analysis
Source: J Clin Med. 2026 May 15;15(10):3827. doi: 10.3390/jcm15103827 (PMC13208049; doi:10.3390/jcm15103827)
Supplement: Supplementary file 1 [file jcm-15-03827-s001.zip › jcm-4231273-supplementary/Supplementary Material 4.pdf]

Table S2. Characteristics of included studies and operative techniques.

Study	Inclusion criteria and mean follow-up	Indication for Collis	Definition of Recurrence	Cohort	Approach	Type of fundoplication			Mesh	Diaphragm relaxing	Redo
						Nissen	Toupet	Other			
McKay et al., 2024	First 50 consecutive patients undergoing elective, primary Lap. PEH repair with Phasix-ST mesh who completed 1 year follow-up<br><br>63.6 ± 3.5 months	Mediastinal dissection failed to achieve 3 cm of intra-abdominal esophageal length	Hernia >2 cm in size on post-operative UGI or EGD	C, n=14	Lap.	3 (21%)	11 (79%)	N/A	14 (100%)	1 (7%)	N/A
				NC, n=13	Lap.	4 (31%)	9 (69%)	N/A	13 (100%)	0	N/A
Pascotto et al., 2022	GERD requiring ARS with ≥2 years follow-up<br><br>104 ± 29.4 months	Short esophagus	N/A	C, n=35	Lap.	N/A	N/A	N/A	0	N/A	34%

				NC, n=261	Lap.	261 (100%)	N/A	N/A	N/A	N/A	19%
Lovece et al., 2022	Recurrent HH with ≥1 year follow-up; emergent cases excluded<br><br>97 ± 56.2 months	Not explicitly mentioned	≥3 cm of stomach above diaphragmatic impression on EGD or UGI	C, n=11	Lap.	5 (45%)	6 (55%)	N/A	0	N/A	100%
				NC, n=31	Lap.	12 (39%)	19 (61%)	N/A	0	N/A	100%
Perrone et al., 2022	Elective robotic HH repair with fundoplication and completed follow-up<br><br>Mean follow-up not	Not explicitly mentioned	N/A	C, n=7	RA	N/A	N/A	N/A	N/A	N/A	N/A

	specified											
				NC, n=13	RA	N/A	N/A	N/A	N/A	N/A	N/A
Lugaresi et al., 2021	Primary minimally invasive ARS for GERD or HH<br><br>98.2 ± 74.5 months	Intra-abdominal esophagus <1.5cm	Recurrence on UGI	C, n=82	VATS-Lap, n=78 (95%)<br>O, n=4 (5%)	82 (100%)	N/A	N/A	N/A	N/A	N/A
				NC, n=213	VATS-Lap, n=206 (97%)<br>O, n=7 (3%)	213 (100%)	N/A	N/A	N/A	N/A	N/A
Lu et al., 2020	Patients who underwent Lap. ARS<br><br>Mean follow-up not specified	Inability for GEJ to rest below diaphragmatic crura after complete mediastinal dissection	Clinically significant recurrence	C, n=214	Lap.	210 (98%)	4 (2%)	N/A	4 (1.9%)	0	28%

				NC, n=798	Lap.	783 (98%)	15 (2%)	N/A	3 (0.4%)	0	5.5%
Bellevue et al., 2018	Patients with short esophagus (<2 cm)<br><br>Mean follow-up not specified	Esophagus <2 cm after mediastinal mobilization	>2 cm on UGI or EGD	C, n=55	Lap.	55 (100%)	N/A	N/A	0	N/A	0
				NC, n=105	Lap.	105 (100%)	N/A	N/A	0	N/A	0
Lugaresi et al., 2016	Left thoracoscopic-laparoscopic Collis-Nissen or standard Nissen<br><br>Mean follow-	Not explicitly mentioned	UGI	C, n=65	VATS-Lap, n=60<br>OL, n=5	65 (100%)	N/A	N/A	N/A	N/A	N/A

	up not specified										
				NC, n=65	VATS-Lap	65 (100%)	N/A	N/A	N/A	N/A	N/A
Durand et al., 2012	Patients undergoing ARS with short esophagus suspected pre-operatively<br><br>43 ± 25.5 months	Radiographic and intra-operative evidence of short esophagus after ≥10 cm of mediastinal mobilization	Not explicitly defined	C, n=11	Lap., n=5 (45.5%)<br>OT, n=5 (45.5%)<br>VATS-Lap, n=1 (9%)	11 (100%)	N/A	N/A	N/A	N/A	N/A
				NC, n=15	Lap., n=13 (87%)<br>OL, n=2 (13%)	N/A	N/A	N/A	N/A	N/A	N/A
Nason et al., 2011	Non-emergent giant PEH (>30% of stomach through hiatus)<br><br>29.3 ± 28.2 months	Not explicitly mentioned	≥10% (or 2 cm) of proximal stomach above the level of the hiatus	C, n=454	Lap., n=451 (99.3%)<br>OL, n=3 (0.7%)	N/A	N/A	N/A	75 (17%)	N/A	N/A

				NC, n=341	Lap., n=331 (97%)<br>OL, n=10 (3%)	N/A	N/A	N/A	31 (9%)	N/A	N/A
Légner et al., 2011	Patients undergoing redo ARS<br><br>Mean follow-up not specified	Unable to achieve 2 cm of intra-abdominal esophageal length	N/A	C, n=16	OT, n=9 (56%)<br>Lap., n=6 (38%)<br>OL, n=1 (6%)	13 (81%)	2 (13%)	Bel., n=1 (6%)	0	N/A	100%
				NC, n=87*	Lap., n=65 (72%)<br>OT, n=13 (15%)<br>OL, n=12 (13%)	44 (49%)	34 (38%)	Dor, n=6 (7%)<br>Bel., n=3 (3%)	0	N/A	100%
Omura et al., 2010	Patients who underwent ARS with sufficient follow-up<br><br>Mean follow-up not specified	Hiatus hernia ≥5 cm	Erosive esophagitis and/or HH identified via EGD and/or UGI	C, n=12	Lap.	12 (100%)	N/A	N/A	0	N/A	N/A

				NC, n=193	Lap.	80 (41%)	113 (59%)	N/A	0	N/A	N/A
Luketich et al., 2010	Elective or urgent Lap. primary repair of giant PEH (>30% stomach herniated into mediastinum)<br><br>33.3 ± 6.3 months	Not explicitly mentioned	Re-herniation of the stomach or wrap identified on UGI	C, n=408	N/A	N/A	N/A	N/A	N/A	N/A	0
				NC, n=239	N/A	N/A	N/A	N/A	N/A	N/A	0
Pirard et al., 2010	Patients undergoing ARS with short esophagus identified by pre-operative UGI<br><br>46 ± 25.1 months	Inadequate esophageal length at time of operation	Intra-thoracic migration on UGI	C, n=27	Lap.	27 (100%)	N/A	N/A	0	0	37%

				NC, n=40	Lap.	40 (100%)	N/A	N/A	0	0	25%
Morino et al., 2006	Elective Lap. Repair of large HH (6 cm on pre-operative EGD, or herniation of ≥50% of stomach into thorax on pre-operative UGI or intra-operative distance of >5 cm between crura)<br><br>43 ± 8.5 months	Short esophagus identified pre-operatively or intra-operatively	Herniation of stomach above level of diaphragm	C, n=14	VATS-Lap	14 (100%)	N/A	N/A	3 (21%)	N/A	N/A
				NC, n=51	VATS-Lap	51 (100%)	N/A	N/A	34 (67%)	N/A	N/A
Chen et al., 2005	Patients with Barrett's esophagus who underwent ARS<br><br>7.4 ± 1.8 months	Not explicitly mentioned	Evidence of mucosal erosion, ulcer, or stricture; or displaced, disrupted, or overtly tight fundoplication on EGD or UGI	C, n=51	OT	51 (100%)	N/A	N/A	N/A	N/A	13.7%

				NC, n=33	OT	33 (100%)	N/A	N/A	N/A	N/A	N/A
Mattioli et al., 2004	Patients undergoing primary ARS<br><br>50 ± 22.4 months	GEJ above the hiatus or 0-3 cm below the hiatus after 7-8 cm mobilization of lower esophagus	HH recurrence or slipping of the fundoplication	C, n=40	OL, n=17 (42.5%)<br>OT, n=11 (27.5%)<br>VATS-Lap, n=10 (25%)<br>Lap., n=2 (5%)	29 (72.5%)	N/A	Bel., n=11 (27.5%)	N/A	N/A	0
				NC, n=130	O, n=68 (52%)<br>Lap., n=62 (48%)	123 (95%)	N/A	Bel., n=7 (5%)	N/A	N/A	0

Mean follow-up time expressed as mean ± SD.
Abbreviations: ARS, antireflux surgery; Bel., Belsey Mark IV; C, Collis; EGD, esophagogastroduodenoscopy; GEJ, gastroesophageal junction; GERD, gastroesophageal reflux disease; HH, hiatal hernia; Lap., laparoscopic; MIS, minimally invasive surgery (not specified laparoscopic or thoracoscopic); N/A, data not available; NC, no Collis; O, open surgery (not specified thoracotomy or laparotomy); OL, open (laparotomy); OT, open (thoracotomy); PEH, paraesophageal hernia; RA, robot-assisted; SD, standard deviation; UGI, upper gastrointestinal series; VATS-Lap, video-assisted thoracoscopic surgery and laparoscopic approach.
*Redo antireflux surgery in this cohort also included three procedures that did not involve a fundoplication, but some demographic information was only available for the whole cohort of 90 patients and hence was included.
