# Supplementary figures and images for: Does the Addition of a Collis Gastroplasty to Antireflux Surgery Reduce Hiatal Hernia Recurrence?: A Systematic Review and Meta-Analysis
Source: J Clin Med. 2026 May 15;15(10):3827. doi: 10.3390/jcm15103827 (PMC13208049; doi:10.3390/jcm15103827)

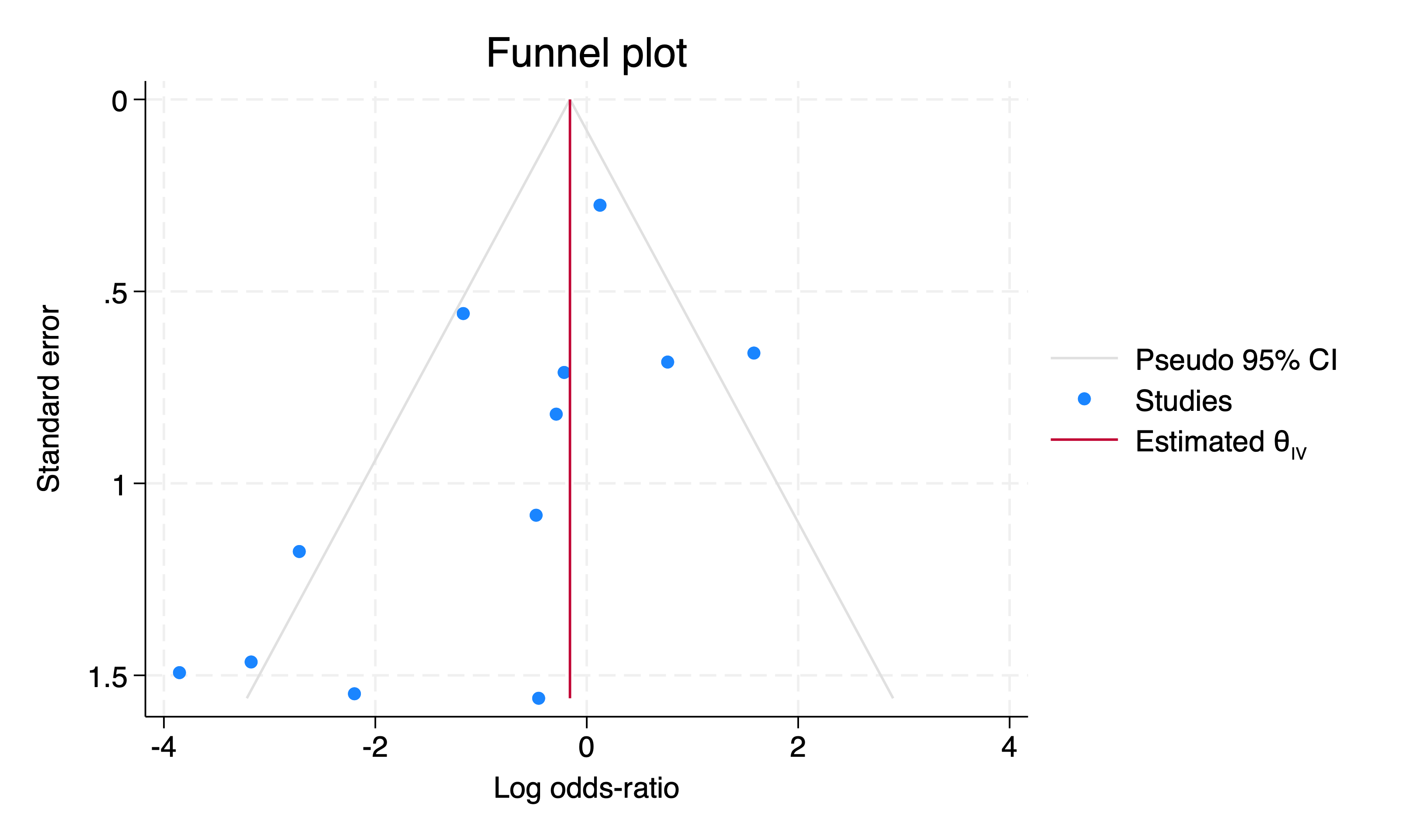

Supplement: Supplementary file 1 [file jcm-15-03827-s001.zip › jcm-4231273-supplementary/Supplementary Material 5a - Recurrence Funnel Plot.tif]

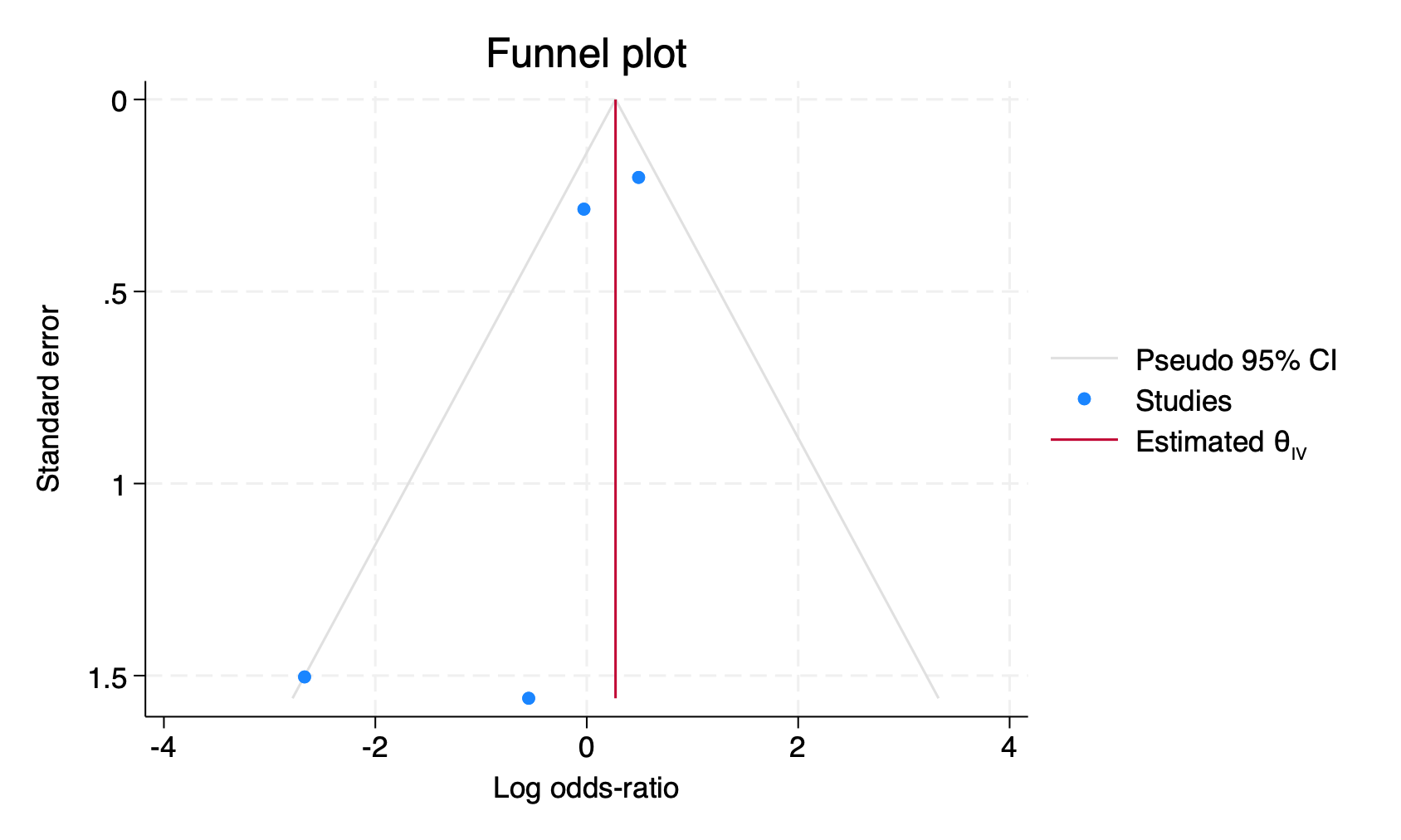

Supplement: Supplementary file 1 [file jcm-15-03827-s001.zip › jcm-4231273-supplementary/Supplementary Material 5b - Dysphagia Funnel Plot.tif]

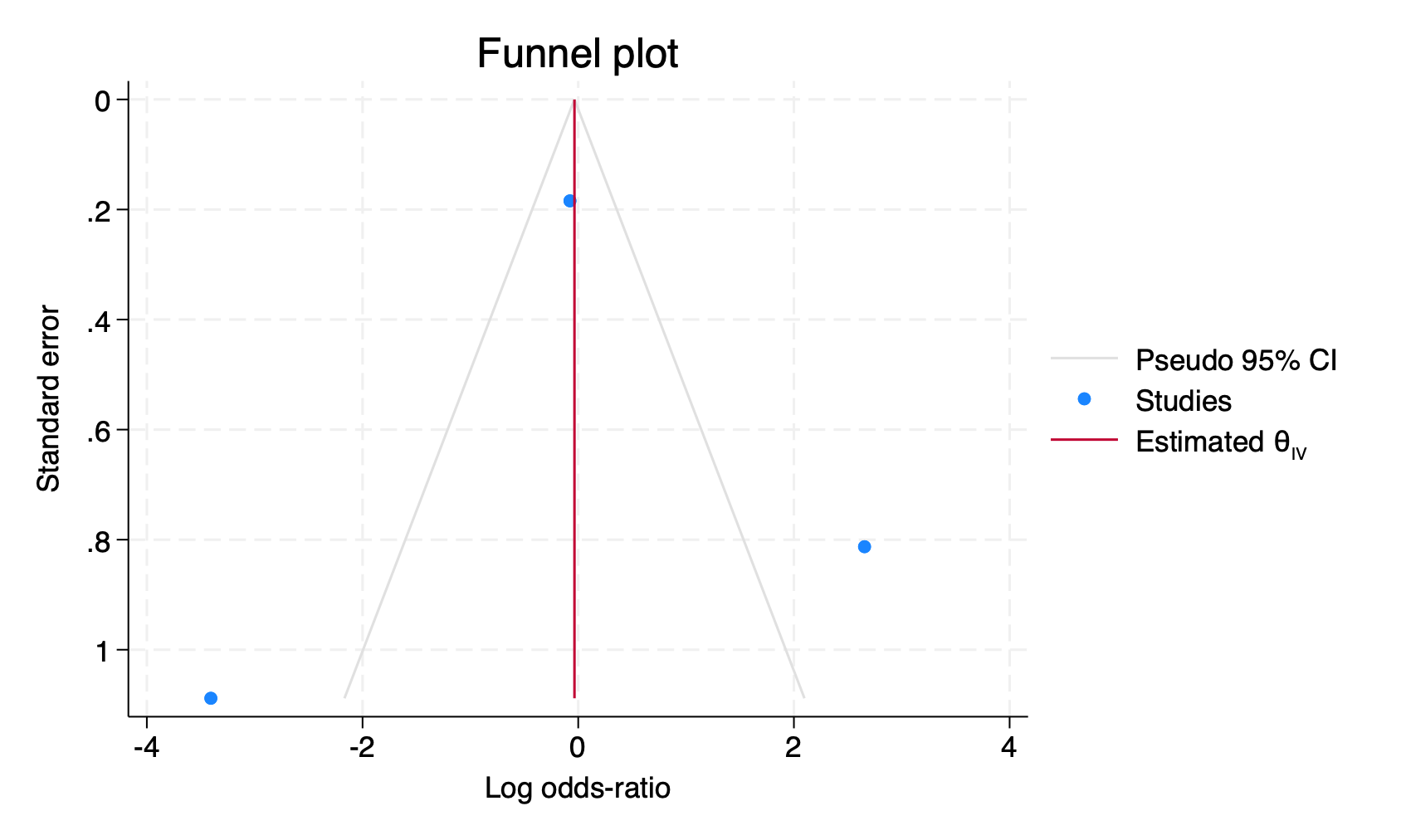

Supplement: Supplementary file 1 [file jcm-15-03827-s001.zip › jcm-4231273-supplementary/Supplementary Material 5c - GERD Funnel Plot.tif]

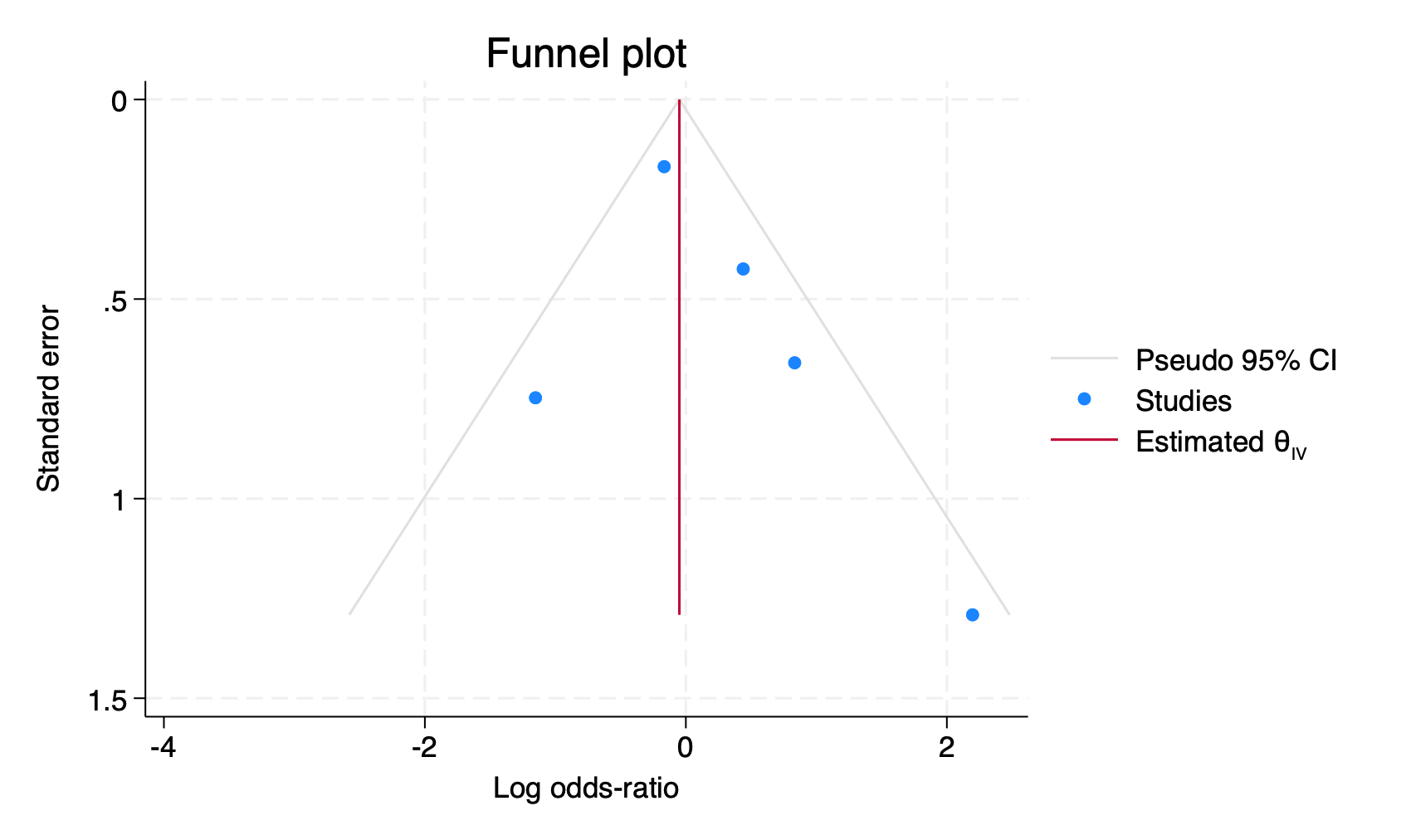

Supplement: Supplementary file 1 [file jcm-15-03827-s001.zip › jcm-4231273-supplementary/Supplementary Material 5d - Medical Anti-Reflux Funnel Plot.tif]

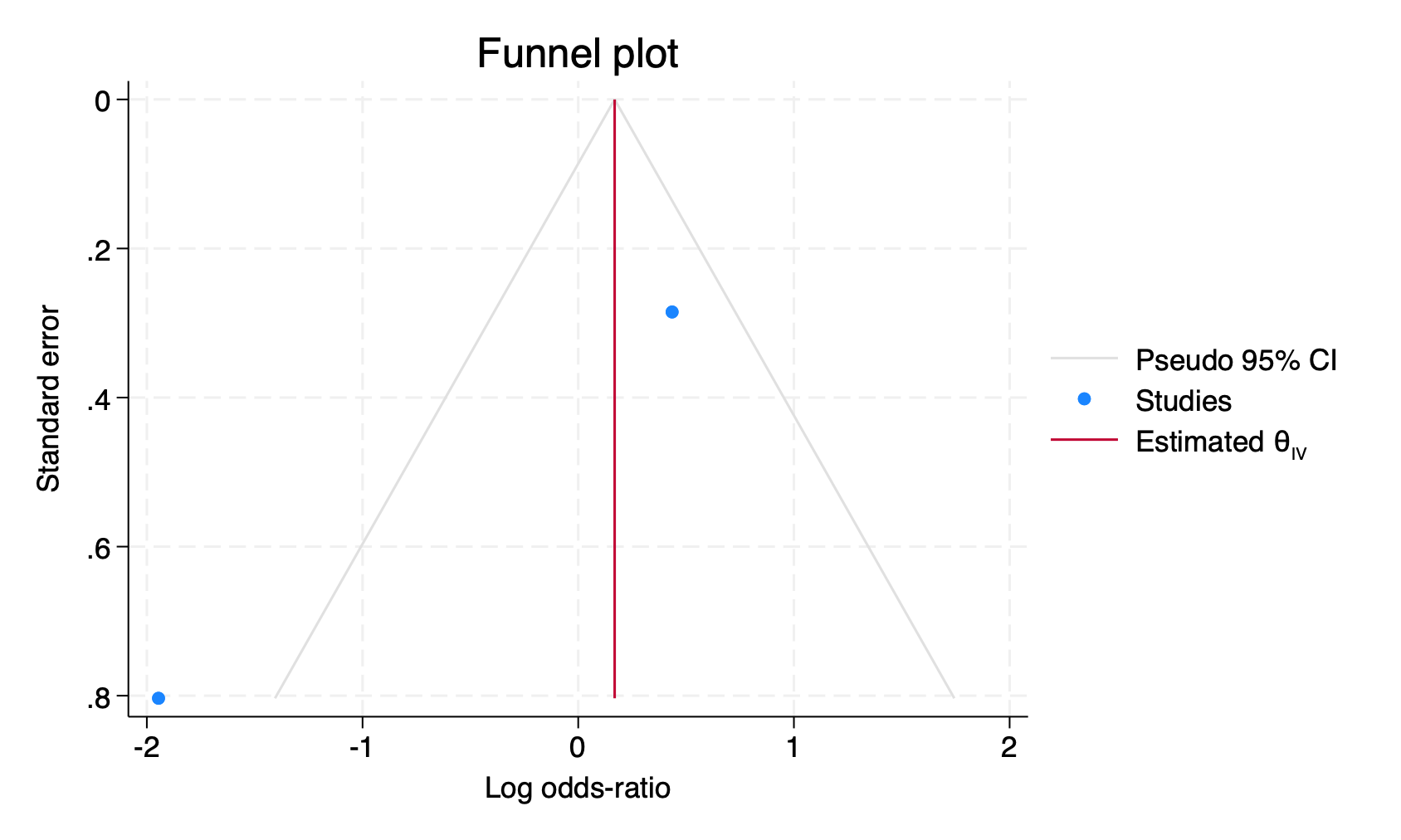

Supplement: Supplementary file 1 [file jcm-15-03827-s001.zip › jcm-4231273-supplementary/Supplementary Material 5e - Regurgitation Funnel Plot.tif]

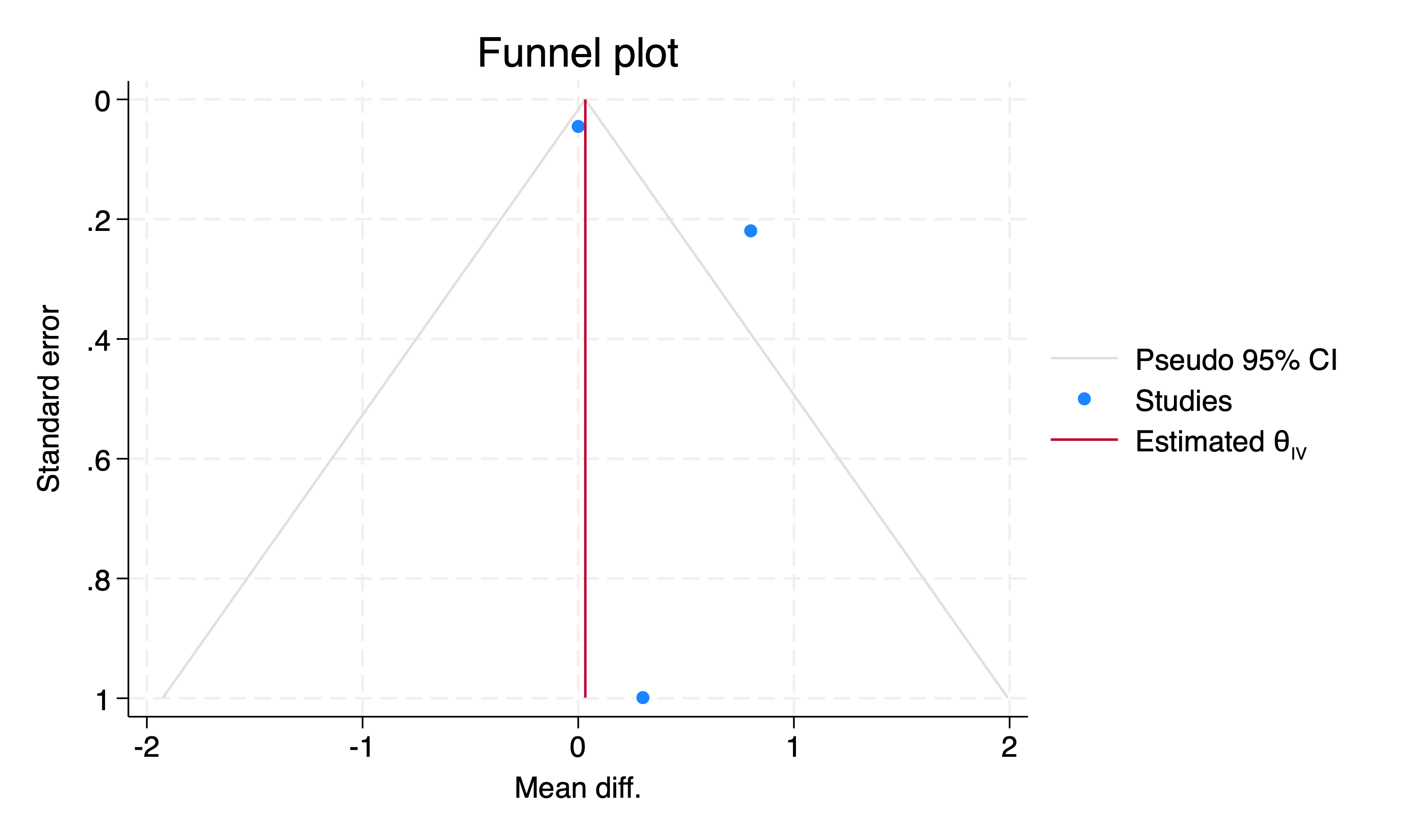

Supplement: Supplementary file 1 [file jcm-15-03827-s001.zip › jcm-4231273-supplementary/Supplementary Material 5f - Length of Stay Funnel Plot.tif]

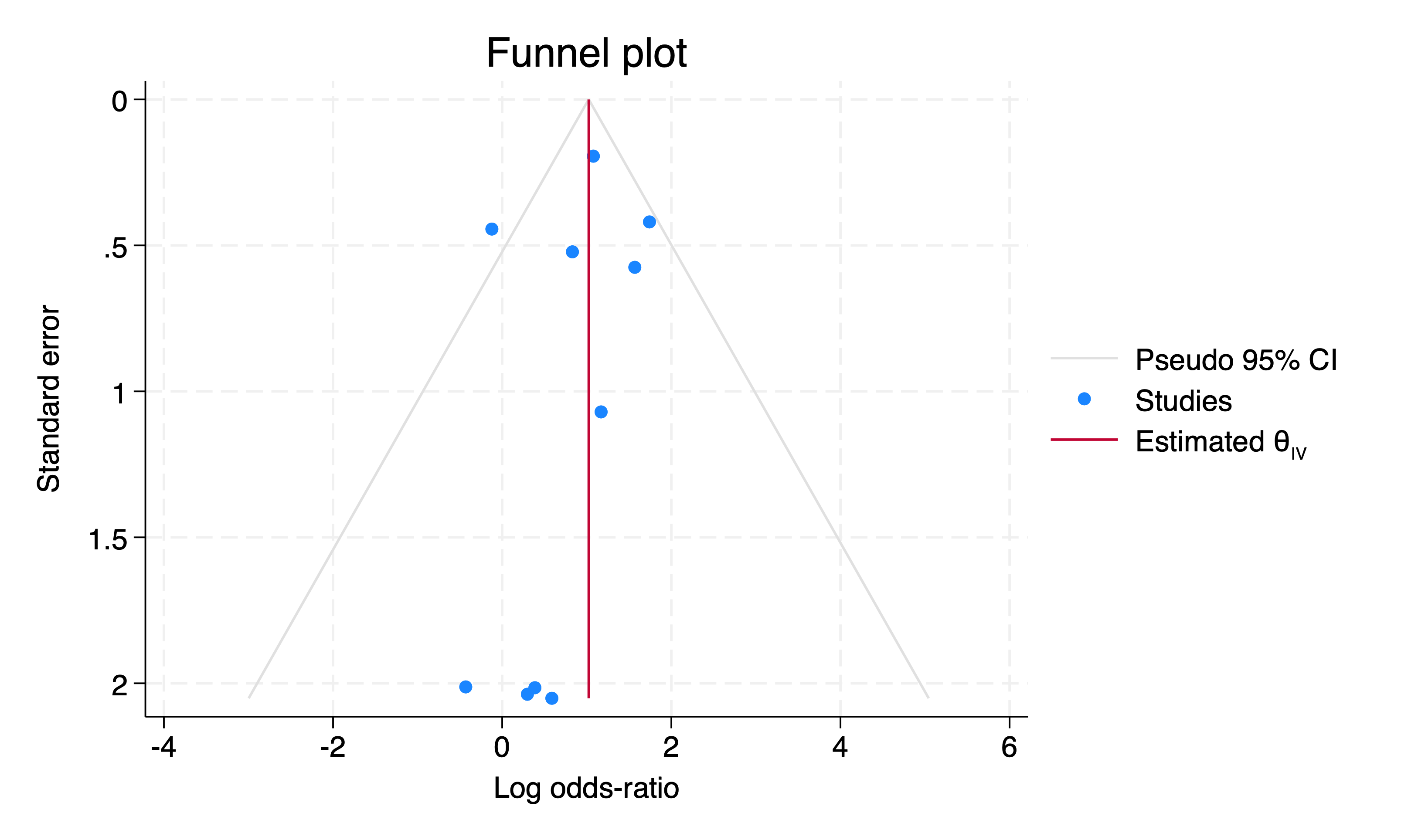

Supplement: Supplementary file 1 [file jcm-15-03827-s001.zip › jcm-4231273-supplementary/Supplementary Material 6a - Complications Funnel Plot.tif]

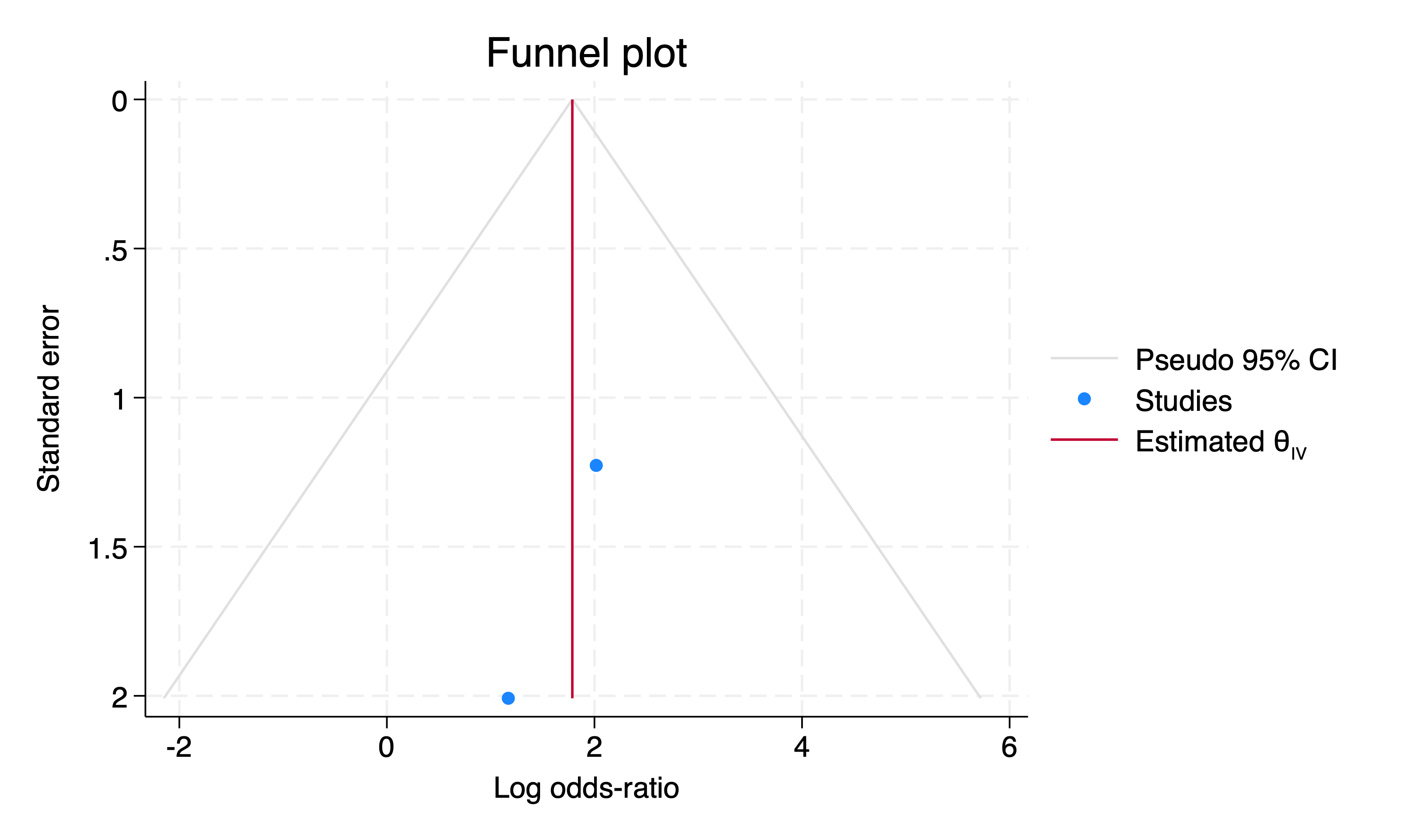

Supplement: Supplementary file 1 [file jcm-15-03827-s001.zip › jcm-4231273-supplementary/Supplementary Material 6b - Abscess Funnel Plot.tif]

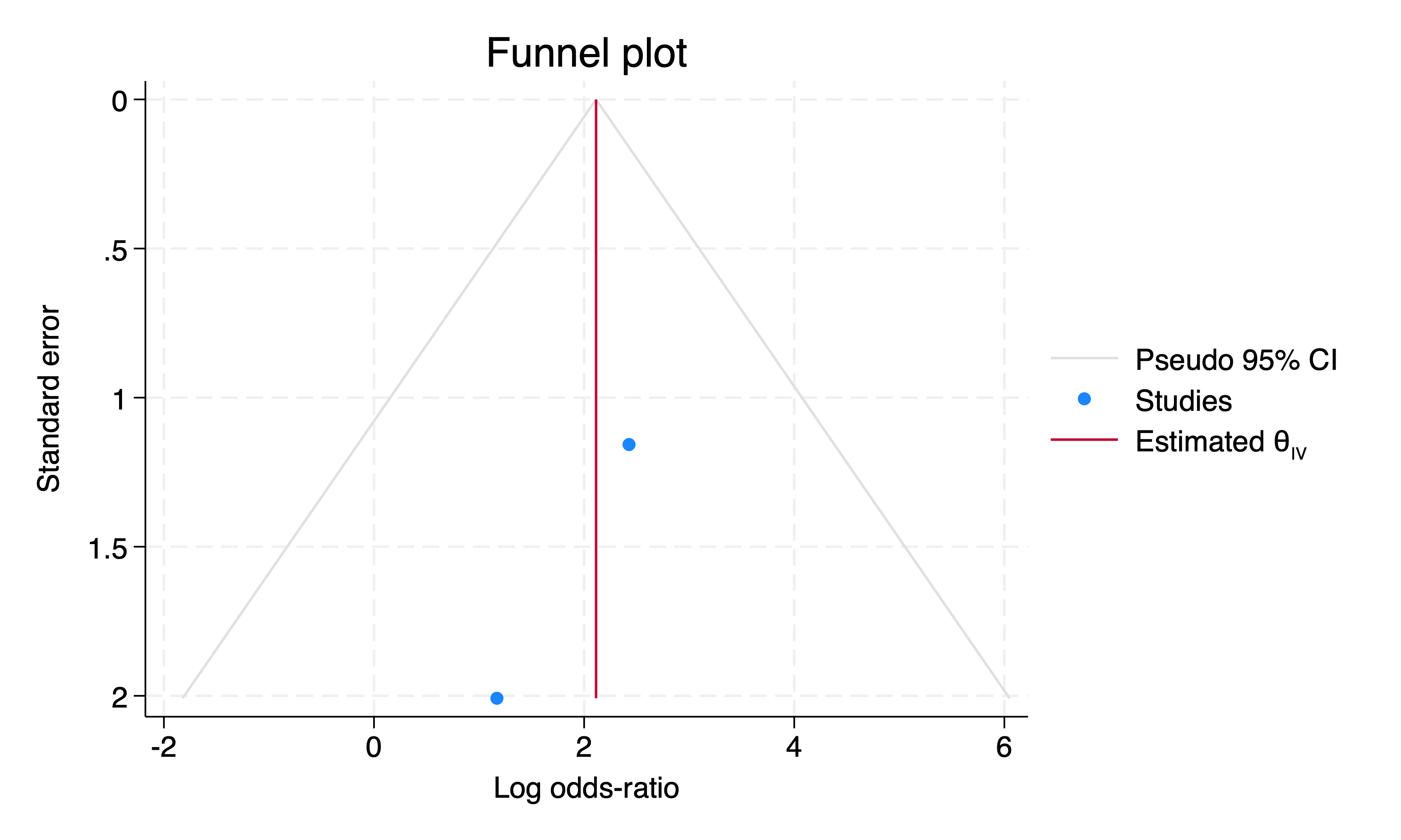

Supplement: Supplementary file 1 [file jcm-15-03827-s001.zip › jcm-4231273-supplementary/Supplementary Material 6c - SSI Funnel Plot.tif]

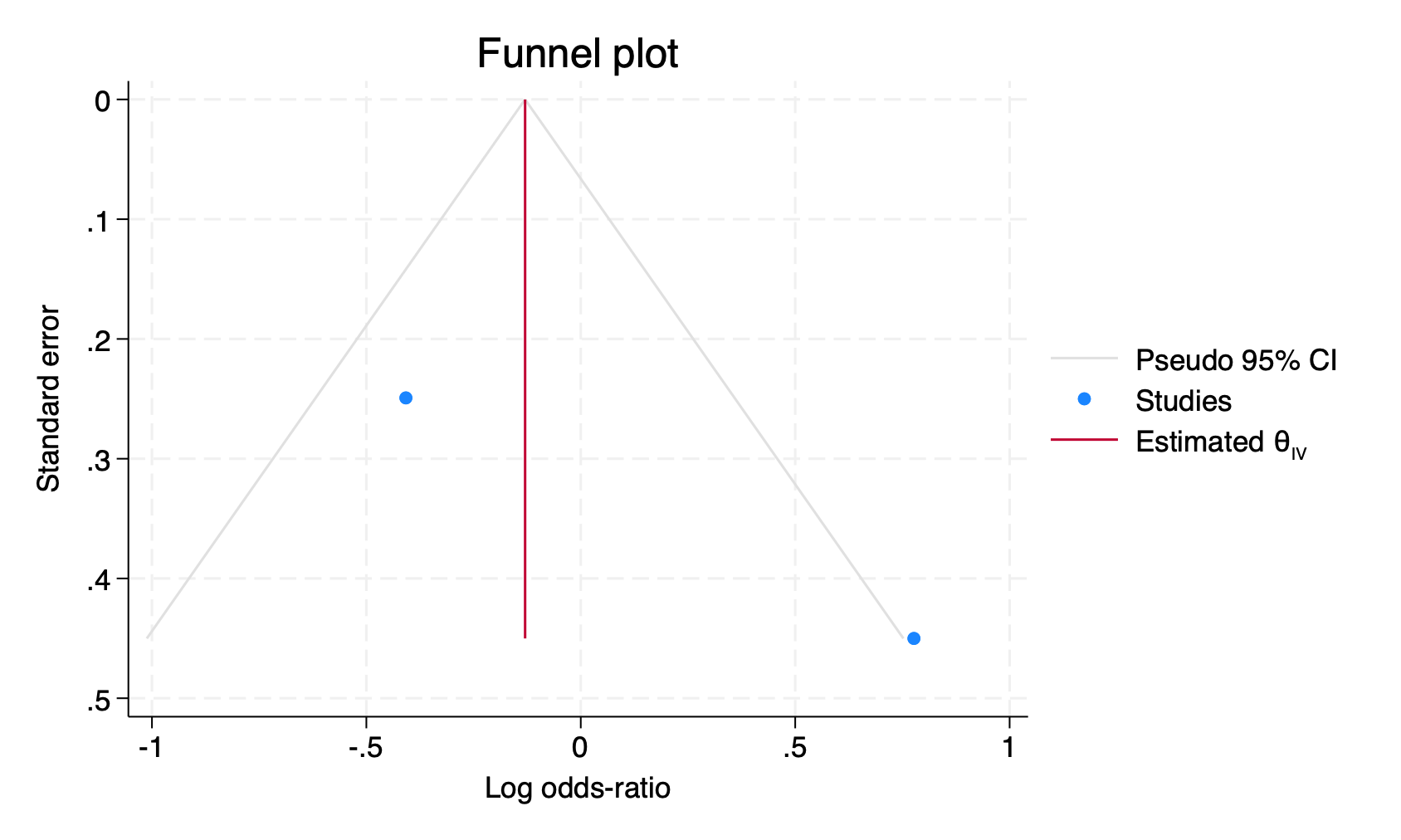

Supplement: Supplementary file 1 [file jcm-15-03827-s001.zip › jcm-4231273-supplementary/Supplementary Material 6d - Readmission Funnel Plot.tif]

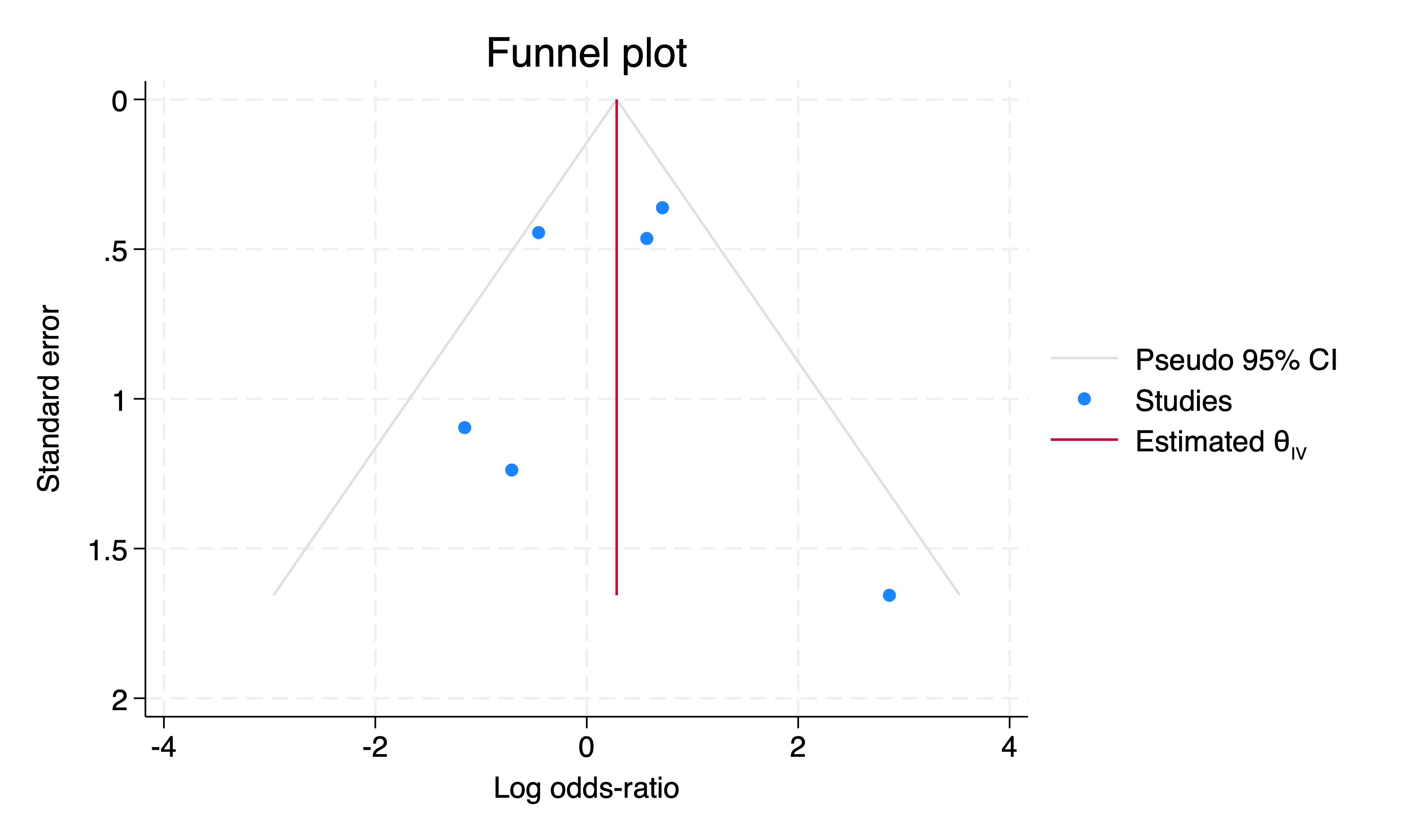

Supplement: Supplementary file 1 [file jcm-15-03827-s001.zip › jcm-4231273-supplementary/Supplementary Material 6e - Reoperation Funnel Plot.tif]

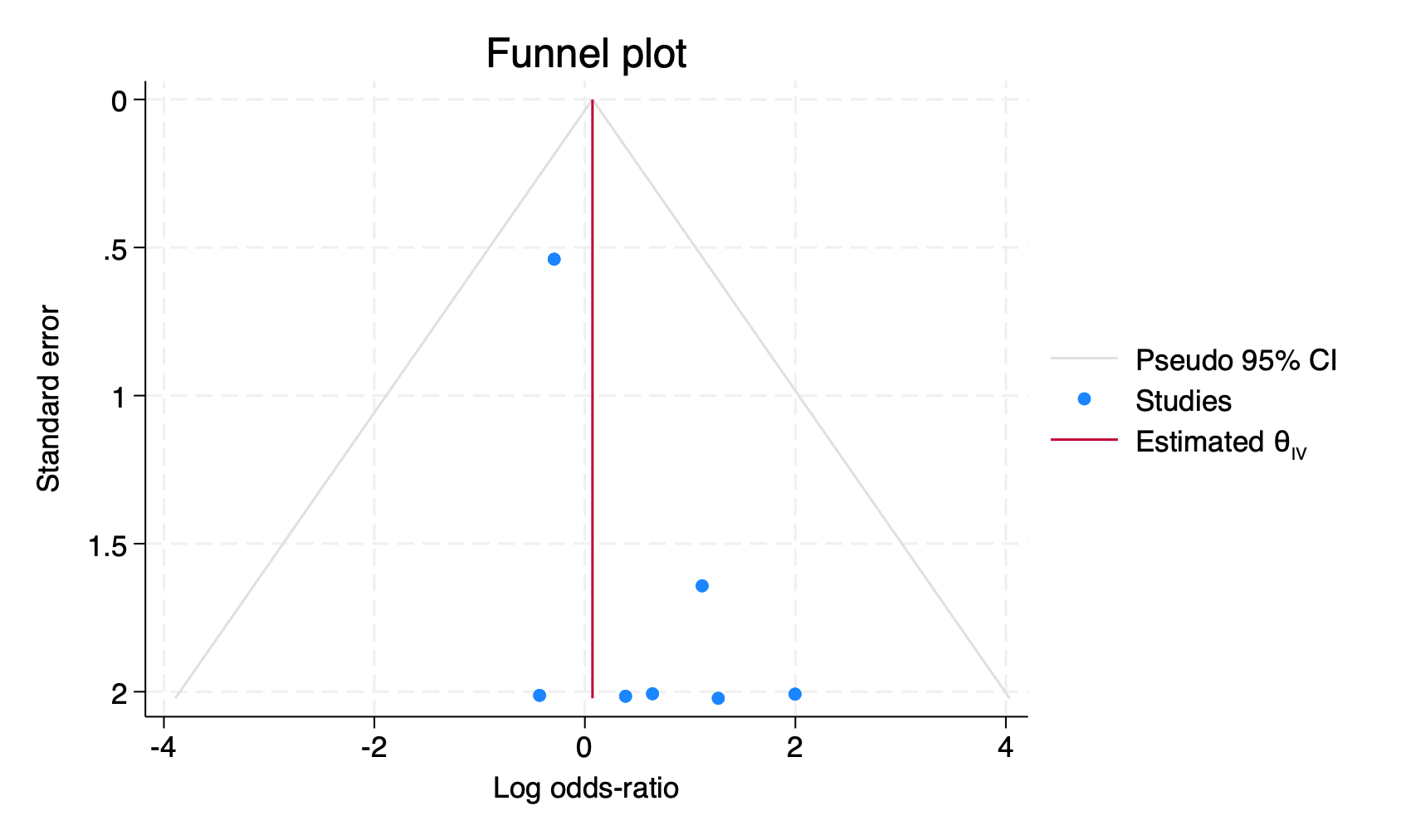

Supplement: Supplementary file 1 [file jcm-15-03827-s001.zip › jcm-4231273-supplementary/Supplementary Material 6f - Mortality Funnel Plot.tif]

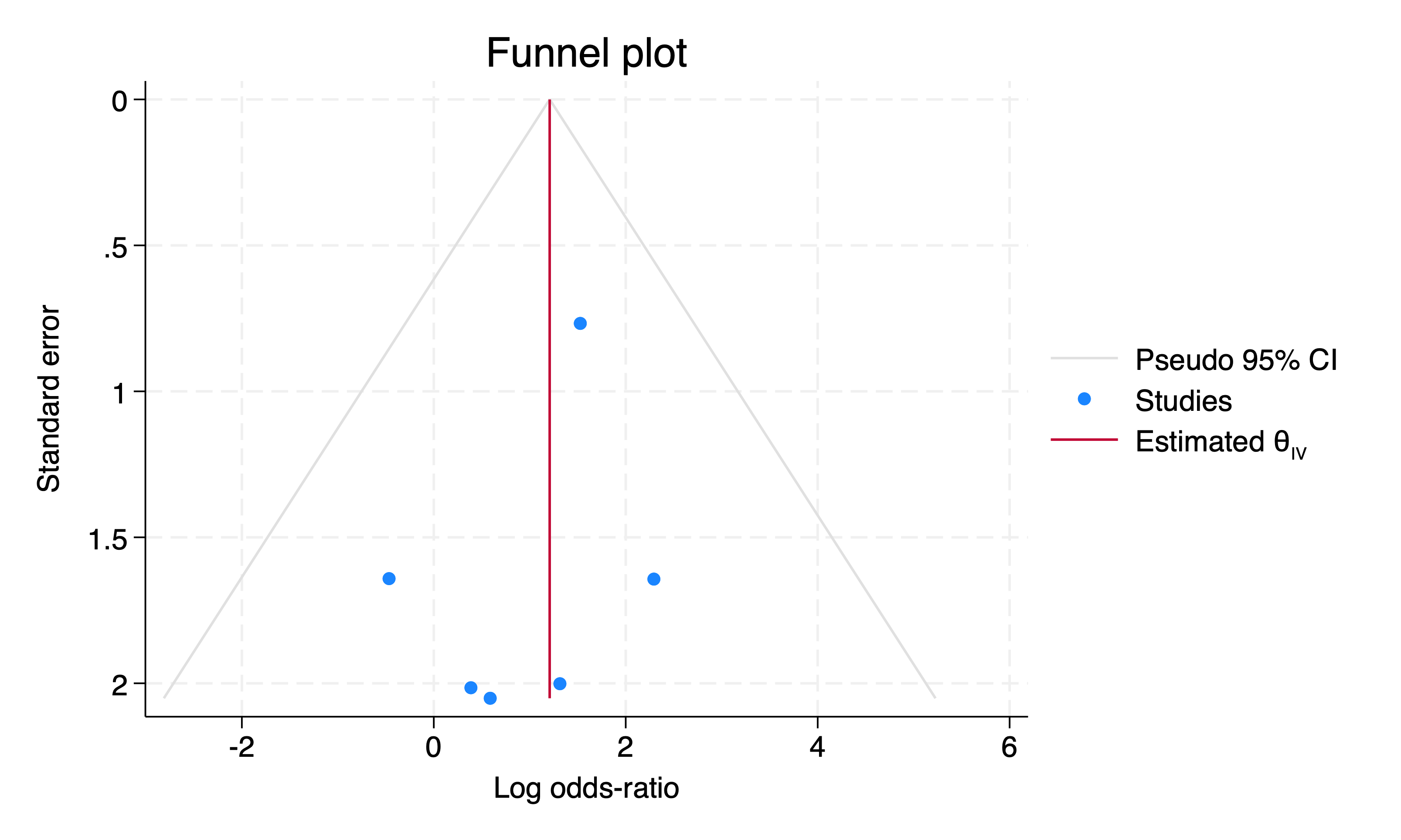

Supplement: Supplementary file 1 [file jcm-15-03827-s001.zip › jcm-4231273-supplementary/Supplementary Material 6g - Leak Funnel Plot.tif]

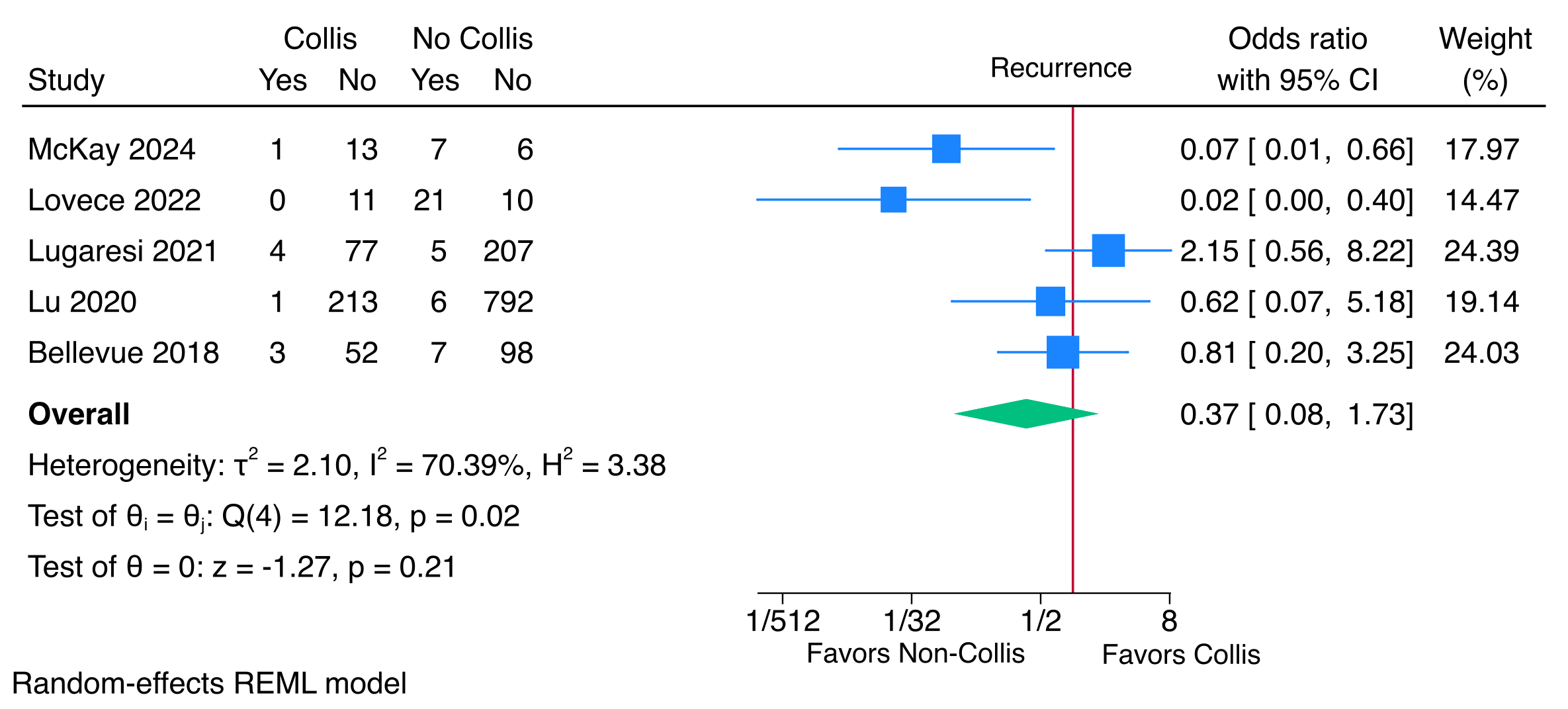

Supplement: Supplementary file 1 [file jcm-15-03827-s001.zip › jcm-4231273-supplementary/Supplementary Material 7a - post2015 forrest.tiff]

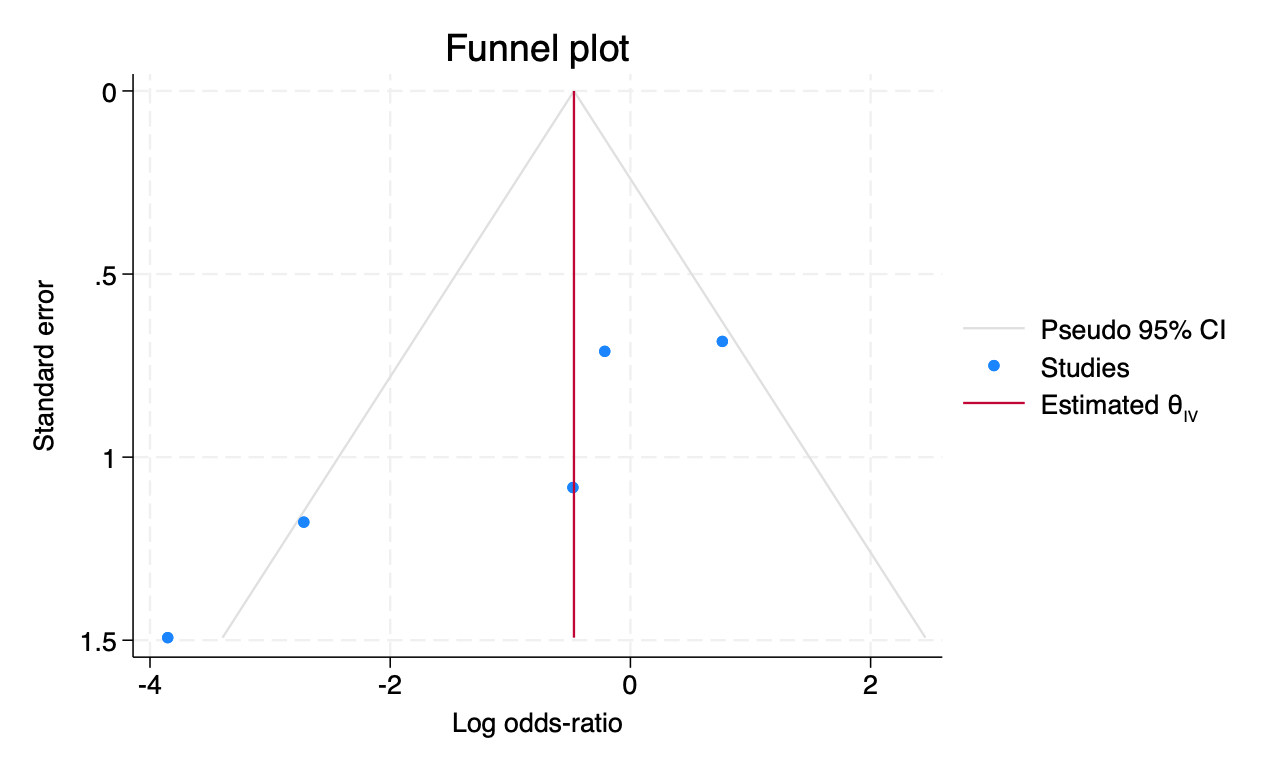

Supplement: Supplementary file 1 [file jcm-15-03827-s001.zip › jcm-4231273-supplementary/Supplementary Material 7b - post2015 funnel.tif]

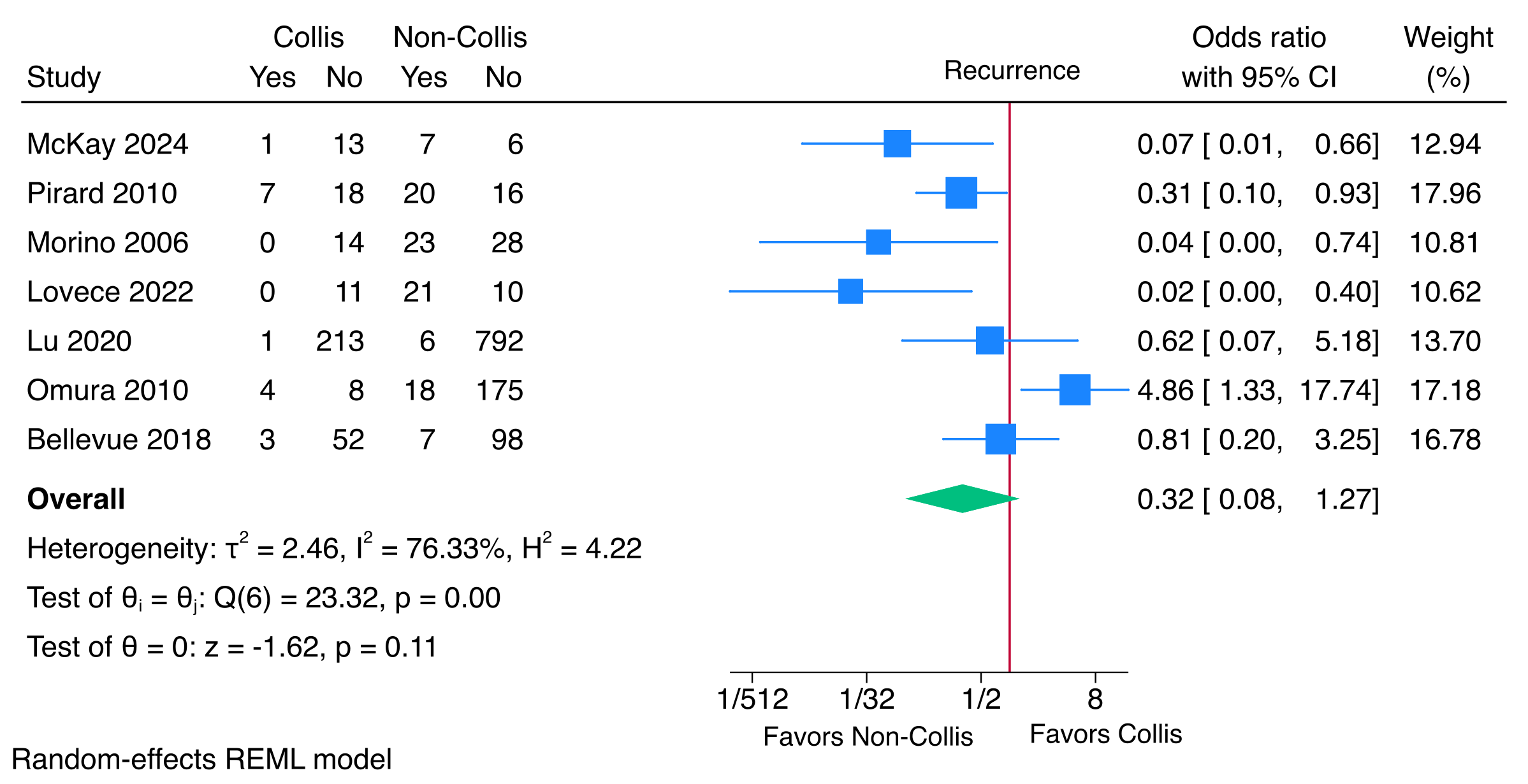

Supplement: Supplementary file 1 [file jcm-15-03827-s001.zip › jcm-4231273-supplementary/Supplementary Material 8a - misonly forest.tiff]

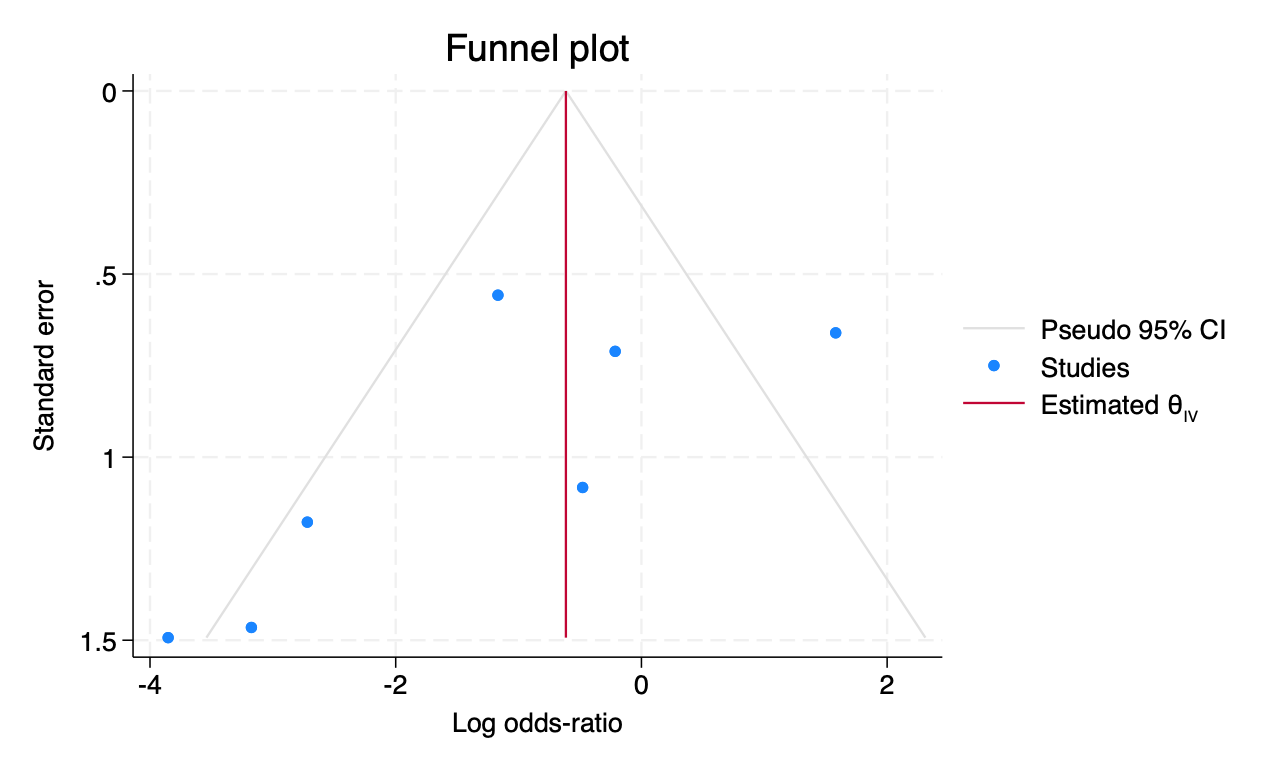

Supplement: Supplementary file 1 [file jcm-15-03827-s001.zip › jcm-4231273-supplementary/Supplementary Material 8b - misonly funnel.tif]

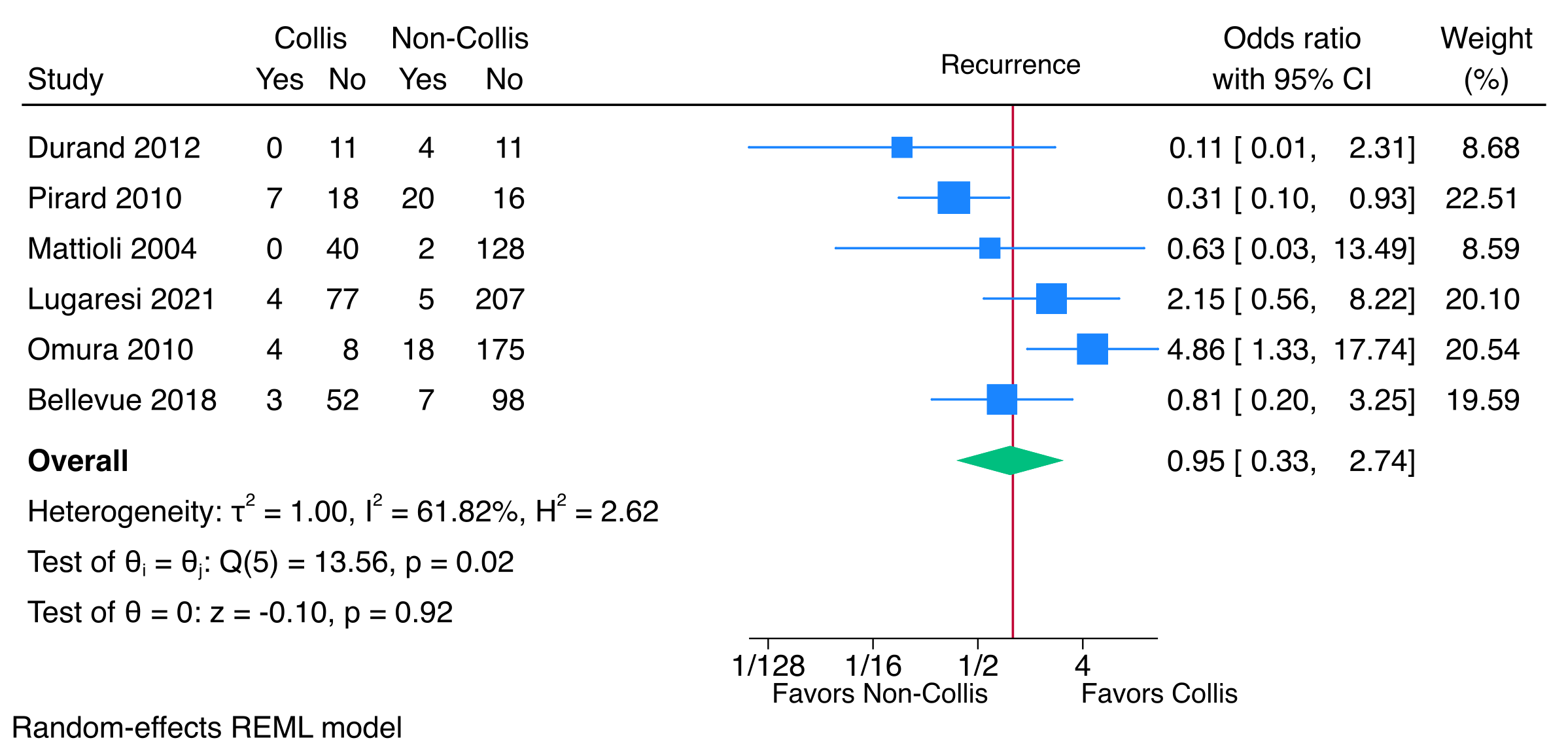

Supplement: Supplementary file 1 [file jcm-15-03827-s001.zip › jcm-4231273-supplementary/Supplementary Material 9a - nomesh forrest.tiff]

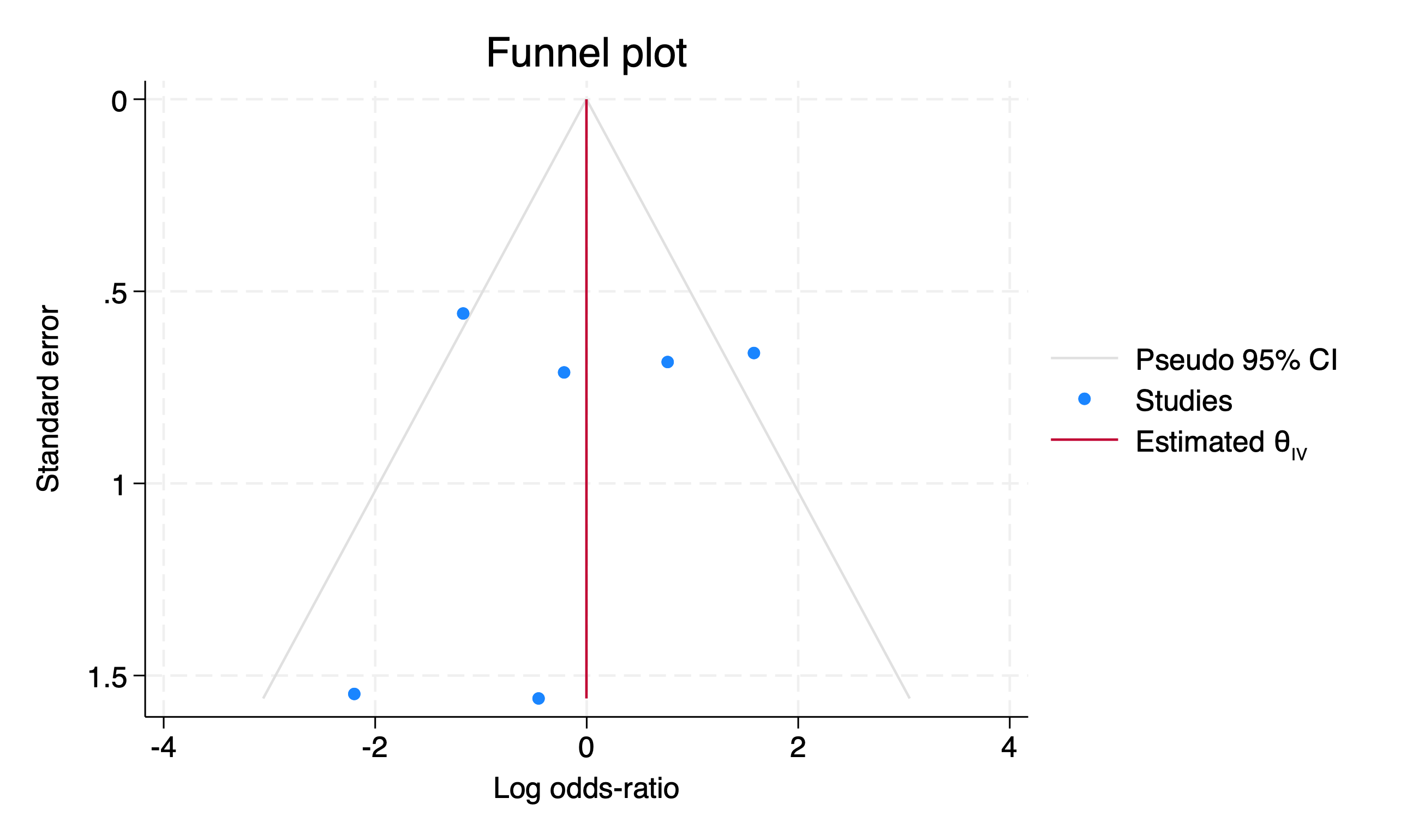

Supplement: Supplementary file 1 [file jcm-15-03827-s001.zip › jcm-4231273-supplementary/Supplementary Material 9b - nomesh funnel.tif]
